# Validation of the sensitivity of the National Emergency X-Radiography Utilization Study (NEXUS) Head computed tomographic (CT) decision instrument for selective imaging of blunt head injury patients: An observational study

**DOI:** 10.1371/journal.pmed.1002313

**Published:** 2017-07-11

**Authors:** William R. Mower, Malkeet Gupta, Robert Rodriguez, Gregory W. Hendey

**Affiliations:** 1 UCLA Department of Emergency Medicine, Ronald Reagan UCLA Medical Center, Los Angeles, California, United States of America; 2 Antelope Valley Hospital Emergency Department, Lancaster, California, United States of America; 3 San Francisco General Hospital, Department of Emergency Medicine, UCSF School of Medicine, San Francisco, California, United States of America; 4 UCSF Fresno, Community Regional Medical Center, Fresno, California, United States of America; Oregon Health and Science University, UNITED STATES

## Abstract

**Background:**

Clinicians, afraid of missing intracranial injuries, liberally obtain computed tomographic (CT) head imaging in blunt trauma patients. Prior work suggests that clinical criteria (National Emergency X-Radiography Utilization Study [NEXUS] Head CT decision instrument [DI]) can reliably identify patients with important injuries, while excluding injury, and the need for imaging in many patients. Validating this DI requires confirmation of the hypothesis that the lower 95% confidence limit for its sensitivity in detecting serious injury exceeds 99.0%.

A secondary goal of the study was to complete an independent validation and comparison of the Canadian and NEXUS Head CT rules among the subgroup of patients meeting the inclusion and exclusion criteria.

**Methods and findings:**

We conducted a prospective observational study of the NEXUS Head CT DI in 4 hospital emergency departments between April 2006 and December 2015. Implementation of the rule requires that patients satisfy 8 criteria to achieve “low-risk” classification. Patients are excluded from “low-risk” classification and assigned “high-risk” status if they fail to meet 1 or more criteria. We examined the instrument’s performance in assigning “high-risk” status to patients requiring neurosurgical intervention among a cohort of 11,770 blunt head injury patients.

The NEXUS Head CT DI assigned high-risk status to 420 of 420 patients requiring neurosurgical intervention (sensitivity, 100.0% [95% confidence interval [CI]: 99.1%–100.0%]). The instrument assigned low-risk status to 2,823 of 11,350 patients who did not require neurosurgical intervention (specificity, 24.9% [95% CI: 24.1%–25.7%]). None of the 2,823 low-risk patients required neurosurgical intervention (negative predictive value [NPV], 100.0% [95% CI: 99.9%–100.0%]).

The DI assigned high-risk status to 759 of 767 patients with significant intracranial injuries (sensitivity, 99.0% [95% CI: 98.0%–99.6%]). The instrument assigned low-risk status to 2,815 of 11,003 patients who did not have significant injuries (specificity, 25.6% [95% CI: 24.8%–26.4%]). Significant injuries were absent in 2,815 of the 2,823 patients assigned low-risk status (NPV, 99.7% [95% CI: 99.4%–99.9%]).

Of our patients, 7,759 (65.9%) met the inclusion and exclusion criteria of the Canadian Head CT rule, including 111 patients (1.43%) who required neurosurgical intervention and 306 (3.94%) who had significant intracranial injuries. In our study, the Canadian criteria for neurosurgical intervention identified 108 of 111 patients requiring neurosurgical intervention to yield a sensitivity of 97.3% (95% CI: 92.3%–99.4%) and exhibited a specificity of 58.8% (95% CI: 57.7%–59.9%). The NEXUS rule, when applied to this same cohort, identified all 111 patients requiring neurosurgical intervention, yielding a sensitivity of 100% (95% CI: 96.7%–100.0%) with a specificity of 32.6% (95% CI: 31.5%–33.6%). Our study found that the Canadian medium-risk factors identified 301 of 306 patients with significant injuries (sensitivity = 98.4%; 95% CI: 96.2%–99.5%), while the NEXUS rule identified 299 of these patients (sensitivity = 97.7%; 95% CI: 95.3%–99.1%). In our study, the Canadian medium-risk rule exhibited a specificity of 12.3% (95% CI: 11.6%–13.1%), while the NEXUS rule exhibited a specificity of 33.3% (95% CI: 32.3%–34.4%).

Limitations of the study may arise from application of the rule by different clinicians in different environments. Clinicians may vary in their interpretation and application of the instrument’s criteria and risk assignment and may also vary in deciding which patients require intervention. The instrument’s specificity is also subject to spectrum bias and may change with variations in the proportion of “low-risk” patients seen in other centers.

**Conclusions:**

The NEXUS Head CT DI reliably identifies blunt trauma patients who require head CT imaging and could significantly reduce the use of CT imaging.

## Abstract 2

In an observational study of over 11,000 blunt trauma head injury patients, William Mower and colleagues validate a decision instrument to identify patients who require computed tomographic imaging.

## Author summary

**Why was this study done?:**

- Physicians, afraid of missing brain injuries, liberally obtain computed tomographic (CT) scanning for head injury patients. CT scans reliably detect injuries, but they are expensive and carry a small risk of radiation-induced lethal cancer.
- We previously developed a decision rule that appears to reliably identify patients with brain injuries, decreasing the need for CT scanning.
- The decision rule assigns head injury patients to low-risk status, and it is therefore safe to omit imaging if they exhibit all the following 8 criteria: no evidence of skull fracture, no scalp hematoma, no neurological abnormalities, normal alertness, normal behavior, no persistent vomiting, normal blood clotting, and age less than 65 years. Patients who are not identified as low risk are automatically assigned high-risk status.
- Before our decision guide can be considered reliable, it needs to be validated in a new large group of patients to ensure that it retains high reliability and still decreases the overall rate of CT scanning.

**What did the researchers do and find?:**

- We requested that emergency physicians from 4 emergency departments in the United States record the presence or absence of the 8 specific criteria, and thereby assess risk status, on every blunt head injury patient they selected for CT scanning.
- 11,770 patients were included in the study, and CT scanning identified brain injuries in 767 patients, including 420 patients who required neurosurgical treatment for their injuries.
- The decision rule assigned high-risk status in all 420 of the patients who required special treatment for their injuries (sensitivity = 100.0%) and in 759 of the patients who had injuries evident on their CT scans (sensitivity = 99.0%).
- Low-risk status was assigned to 2,815 patients who had neither brain injury on CT nor required neurosurgical care.

**What do these findings mean?:**

- Our decision guide reliably identified patients who required neurosurgical treatment of their injuries, as well as those who had brain injuries evident on CT scan.
- The decision guide correctly identified nearly one-quarter of all patients as being at low risk for injury and safe to exclude from scanning.
- Clinical use of our decision guide will allow physicians to reliably identify patients with serious brain injuries, while simultaneously decreasing the number of patients who require CT scanning.

## Introduction

It is estimated that each year in the United States, over 4.8 million patients present to emergency departments (EDs) after sustaining blunt head trauma. Head computed tomographic (CT) imaging is performed in 3.9 million of these patients, of which 400,000 (10%) are positive for any traumatic abnormality [1].

The evaluation of blunt head injury patients can be challenging. Unrecognized injuries can result in permanent brain damage, severe disability, and even death.

Noncontrast head CT provides a reliable means of evaluating closed head injury patients, but CT imaging is expensive and exposes patients to a small risk of lethal malignant transformation [2–4]. Furthermore, though ED visits for traumatic brain injury (TBI) have increased in the last decade, the prevalence of significant injury remains low and unchanged, despite increases in head CT utilization [5–8]. Because most patients receive little or no benefit from imaging, while bearing the expense and radiation exposure, there has been a national push towards more selective use of head CTs in adult blunt head injury patients who are thought to be at low risk for significant injury [9,10].

Prior work suggests that clinical decision instruments (DIs) can identify blunt head injury patients who have very low risk of significant intracranial injuries and for whom CT imaging can be safely omitted [8,11–14]. This previous work is limited by the fact that many of the rules apply only to subsets of blunt trauma patients, have yet to be adequately validated, or lack validation with sufficient precision to ensure reliability [8,11–15]. For example, while the New Orleans and Canadian Head CT rules exhibit sensitivities near 100%, their associated lower confidence limits indicate these rules could still misclassify nearly 5% of patients requiring neurosurgical intervention [11,14,16–18].

The purpose of this study is to assess the validity of a previously derived DI (the National Emergency X-Radiography Utilization Study [NEXUS] Head CT instrument) that can be applied to essentially all adult blunt head injury patients [8]. The instrument was developed in an observational study involving 13,728 patients, including 917 with serious injuries, and required participating clinicians to prospectively assess blunt head injury patients for the presence or absence of specific criteria. We applied recursive partitioning to identify 8 of the criteria that predicted intracranial injuries with high sensitivity while retaining the highest specificity. These criteria form the NEXUS head imaging DI. The current assessment was specifically designed to validate this instrument in a new cohort, with sufficient precision in the measurement of the lower confidence limit for sensitivity, to ensure reliability [15].

A secondary goal of the study was to complete an independent validation and comparison of the Canadian and NEXUS Head CT rules among the subgroup of patients meeting the inclusion and exclusion criteria of the Canadian rule.

## Methods

### Ethics statement

Using a multicampus review mechanism, we obtained institutional review board approval from the University of California, Los Angeles (UCLA) Committee on Human Research for the 2 University of California sites. We obtained separate approvals from the Antelope Valley Hospital and UCSF Fresno, Community Regional Medical Center institutional review boards for their respective participating institutions. Because the study did not alter patient care, or present more than minimal risk, and because many patients would be critically ill, intoxicated, or have neurological impairments rendering them incapable of providing consent, we obtained a waiver of informed consent at all sites. We controlled all aspects of the study design, implementation, analysis, and manuscript preparation without influence from the grant funding agencies.

### Participating centers

We conducted a prospective observational study of consecutive blunt head injury patients presenting to 4 general EDs (Antelope Valley Hospital, Lancaster, California; San Francisco General Hospital, San Francisco, California; UCLA Ronald Reagan Medical Center, Los Angeles, California; UCSF Fresno Community Regional Medical Center, Fresno, California). Participating institutions were specifically chosen to provide experience from university and community hospitals, centers with and without residency programs, public and private facilities, and exposure to a broad range of patients (children, adults, and the elderly) and environments, including urban, suburban, and rural communities. We conducted the study between April 2006 and December 2015, and the full protocol is included as a supplementary file to this article (S1 Protocol).

The study was designed to assess the validity of the following 8 criteria (the NEXUS Head CT DI) to exclude intracranial injuries in blunt head injury patients: no evidence of skull fracture, no scalp hematoma, no neurosurgical deficits, normal level of alertness, normal behavior, no persistent vomiting, no coagulopathy, and age less than 65 years.

These criteria were identified in our prior derivation study as indicators for potential candidates for our NEXUS head imaging DIs [8]. Box 1 presents the detailed description of each criterion we provided to the treating clinicians. We considered patients to be at low risk of intracranial injuries and safe for omission of CT head imaging if all 8 criteria were absent. We regarded patients who exhibited 1 or more of the criteria, and those with 1 or more unassessed criteria, to be at high risk for intracranial injury and in need of CT imaging.

**Box 1. Conditions that must be absent for low-risk classification by the National Emergency X-Radiography Utilization Study (NEXUS) Head computed tomographic (CT) instrument**

Terms are defined for purposes of clarity and to ensure consistent data collection.

**Evidence of skull fracture includes** signs of **basilar skull fracture**, including, but are not limited to, periorbital or peri-auricular ecchymoses, hemotympanum, and drainage of clear fluid from the ears or nose. Signs of **depressed or diastatic skull fracture** include a palpable step-off of the skull, a stellate laceration from a point source, or any injury produced by an object striking a localized region of the skull (such as a baseball bat, club, pool cue, golf-ball, baseball, pipe, etc.).

**Scalp hematoma** refers to swelling secondary to hematoma formation over any portion of the bony calvarium. Injuries that do not involve the calvarium, including hematomas limited to the face and neck, are not considered scalp hematomas.

**Neurologic deficit** refers to any abnormal neurologic finding revealed by detailed testing. This may include motor or sensory deficits (abnormal weakness or sensation in any 1 or more of the 4 extremities, as determined by systematic testing of muscle strength and sensation in all 4 limbs), cranial nerve abnormality (particularly cranial nerves II through XII, as determined by systematic testing of each nerve), cerebellar abnormality as manifested by ataxia, dysmetria, dysdiadokinesis, or other impairment of cerebellar function (as determined by systematic testing of cerebellar function, including tests of ataxia, and finger-nose-finger, heel-to-shin, and rapid alternating movements), gait abnormality or inability to walk normally (may be due to inadequate strength, loss of balance, or ataxia; it is determined by systematic testing of gait, including tandem and heel-to-toe walking and Romberg testing), or any other impairment of neurological function.

**Abnormal level of alertness** is evidenced by a variety of findings, including, but not limited to, a Glasgow coma score of 14 or less; delayed or inappropriate response to external stimuli; excessive somnolence; disorientation to person, place, time, or events; inability to remember 3 objects at 5 minutes; perseverating speech; and other neurological impairments.

**Abnormal behavior** is any inappropriate action displayed by the victim. It includes such things as excessive agitation, inconsolability, refusal to cooperate, lack of affective response to questions or events, and violent activity.

**Persistent vomiting** is evidenced by recurrent (more than 1 episode) projectile or forceful emesis (either observed or by history) after trauma.

**Coagulopathy** is any impairment of normal blood clotting such as that which occurs in hemophilia, secondary to medications (Coumadin, heparin, aspirin, etc.), hepatic insufficiency, and other conditions.

**Age 65 years or more** is determined by available history.

An emergency physician at each center served as a study liaison and was charged with training participating clinicians in the criteria definitions and conduction of the study. Liaisons ensured that criterion assessments, radiographic results, and final outcomes were collected on all enrollees.

### Patients

The study population consisted of all acute blunt head trauma patients undergoing CT head imaging at the participating centers. We enrolled patients when the treating provider ordered CT head imaging. We excluded patients with penetrating trauma, those with delayed presentations (greater then 24 hours after injury), and patients undergoing imaging for reasons unrelated to trauma. We also excluded patients who were transferred into a participating center with known intracranial injuries. To maximize compliance, participating centers adopted a protocol whereby CT imaging would not be performed until decision criteria had been assessed and recorded [19]. We cautioned clinicians against using the DIs as the sole determinant in making imaging decisions. The ultimate decision to obtain or omit imaging was made at the discretion of the treating provider and was not dictated by the study protocol.

### Data collection

At the time of enrollment, the treating clinician collected and recorded limited demographic information (date of birth, sex, race, and ethnicity) and documented whether DI criteria were present, absent, or could not be assessed (for example coagulopathy in a comatose patient). In implementing the DI, we considered criteria that could not be assessed to be abnormal and excluded the patient from low-risk classification. This approach maximized safety, ensuring that low-risk assignments were based on actual measured assessments rather than missing information.

Physicians had the option to bypass data collection and obtain immediate imaging, prior to criterion assessment, on any patient they felt might be harmed by even minimal delay. Such patients were labeled as “unstable,” and clinicians were instructed to complete assessments of the criteria as soon as possible—and before imaging results were available. We considered “unstable” patients to be excluded from low-risk classification.

We also collected assessments for the Canadian Head CT rule, including inclusion criteria (initial Glasgow Coma Scale [GCS] score of 13 or greater, and injury within the last 24 hours), exclusion criteria (age less than 16 years, minimal head injury, no clear history of trauma as the primary event, obvious penetrating skull injury or depressed skull fracture, an acute focal neurological deficit, seizure prior to assessment, bleeding disorder or using anticoagulants, returned for reassessment of the same head injury), and risk criteria (High-risk criteria [for neurosurgical intervention]: GCS score <15 two hours after injury, suspected open or depressed skull fracture, any sign of basal skull fracture, 2 or more episodes of vomiting, and age ≥65 years. Medium-risk criteria [for significant brain injury]: Amnesia before impact >30 minutes, and dangerous mechanism).

### Outcome measures

We defined our primary outcome a priori as the need for neurosurgical intervention, defined specifically as 1) death due to head injury, 2) need for craniotomy, 3) elevation of skull fracture, 4) intubation related to head injury, or 5) intracranial pressure monitoring, within 7 days of head injury [14].

Our secondary outcome was clinically significant head injury evident on CT imaging, as defined by Stiell, et al [14]. This included all injuries evident on CT head imaging except for the following in neurologically intact individuals: solitary small contusions, localized subarachnoid hemorrhage less than 1 mm thick, thin subdural hematomas less than 4 mm thick, isolated pneumocephaly, and closed depressed skull fractures that did not violate the inner table [14].

Formal radiographic interpretations and outcome assignments were all completed without knowledge of the criteria assessments recorded for each patient. We assigned each patient to the following 3 final outcome classes: 1) no significant injury, 2) significant injury (including injuries requiring neurosurgical intervention), and 3) injury requiring neurosurgical intervention. Two separate reviewers completed outcome assessments for each patient, with a third reviewer assigning outcomes in instances where the 2 initial assignments were discordant.

### Assessing the potential for verification bias

Because we only enrolled patients undergoing head imaging, it is possible that important injuries may have gone unrecognized among the unimaged patients. To address the potential for verification bias that might arise from this approach, we conducted 3-month follow-up interviews on 368 consecutive blunt head injury patients who presented between July 2011 and March 2015. Patients were eligible for inclusion in this study if they had been evaluated in the UCLA ED for blunt head injury, did not receive head imaging as part of their evaluation, and agreed to participate in a 3-month follow-up interview and medical record review, and provide written informed consent. Patients were excluded from the study if they were not evaluated for blunt head injury during the enrollment period, underwent head CT imaging as part of their initial evaluation, or refused to participate in a 3-month follow-up interview and medical record review, or provide written informed consent. The size of this cohort reflects our desire to estimate the potential for verification bias to within 1.0%. In follow-up interviews, each patient was asked whether they had received radiographic head imaging elsewhere during a subsequent visit, and if so, the type of imaging (CT, MRI, skull films). Each patient was also assessed to determine whether they were diagnosed with intracranial injuries or required neurosurgical intervention. We also conducted a review of case logs and trauma logs to identify any instances of significant intracranial injuries or injuries requiring neurosurgical intervention that occurred among head injury patients who were seen on their initial presentation but not imaged.

### Statistical analysis

#### Sample size estimation

To be clinically reliable, DIs that guide imaging of blunt head injury patients must satisfy 2 requirements. First, every blunt head injury patient with an injury requiring neurosurgical intervention must exhibit at least 1 risk criterion. Thus, the ideal instrument must exhibit a sensitivity of 100%. Second, patients identified as risk-free by the instrument must never harbor intracranial injuries requiring intervention. Thus, the instrument must exhibit a 100% negative predictive value (NPV).

Verifying sensitivity and NPV value at absolute levels is statistically and pragmatically impossible, but it is possible to estimate the lower confidence levels for these proportions to a 99.0% level. At this level of precision, the risk of adverse outcome due to missed injuries approaches the risk of lethal malignant transformation secondary to the radiation exposure that would occur from additional imaging [3,15,20].

Validating a 95% lower confidence bound of 99.0% for a measured sensitivity of 100.0% requires evaluations on 368 patients having injuries that require neurosurgical intervention. Similar validation of the NPV requires assessments on 368 patients assigned low-risk classification [21]. Because patients assigned low-risk classification are more prevalent than those requiring neurosurgical intervention, our final sample estimate was driven by the need to enroll 368 patients who required neurosurgical intervention.

#### Data analysis

Using standard formulae, we calculated the screening performance of the NEXUS Head CT DI. Our primary outcome was the point measure and confidence interval (CI) for the sensitivity in detecting injuries that require neurosurgical intervention. We also determined point measures and CIs for the associated NPV and specificity and assessed the DI’s sensitivity, NPV, and specificity in detecting significant injuries.

A secondary goal of our study was the completion of a planned comparison of the NEXUS and Canadian Head CT instruments. To achieve this goal, we identified the subset of patients who satisfied the inclusion and exclusion criteria of the Canadian Head CT rule. We then calculated the operator characteristics and associated CIs among patients in this cohort for the Canadian Head CT “high-risk” rule (designed to identify patients requiring neurosurgical intervention), the “medium-risk” rule (designed to identify patients with clinically important brain injury), and the NEXUS Head CT rule.

## Results

**Fig 1:**
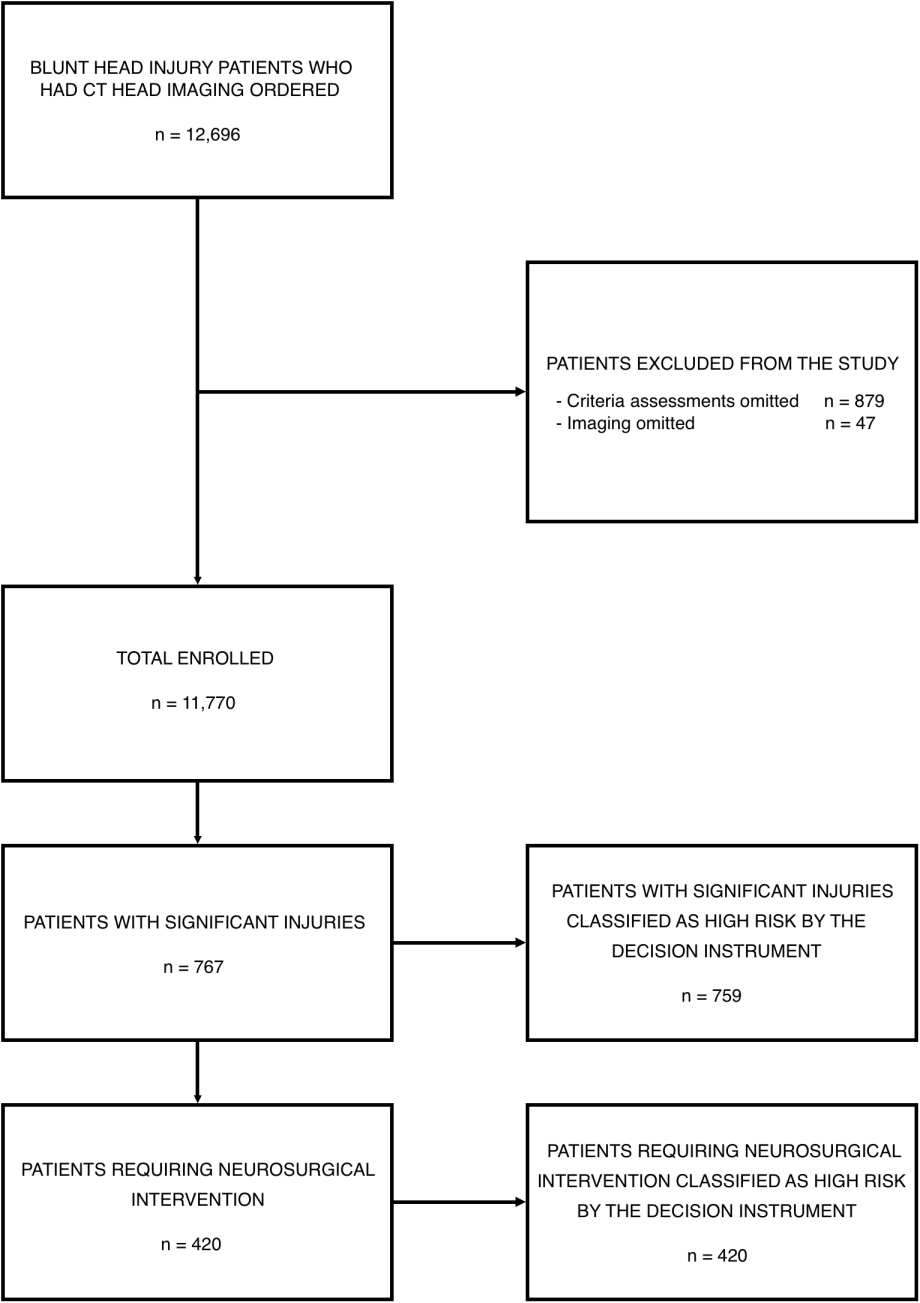
Patient enrollment in the National Emergency X-Radiography Utilization Study (NEXUS) Head computed tomographic (CT) validation trial.

Between April 18, 2006 and December 10, 2015, physicians ordered CT head imaging on 12,696 patients. Criteria assessments were completed for 11,817 of these patients (93.1%), and imaging was completed in 11,770 patients who form the enrollment cohort, including 420 (3.6%) patients who required neurosurgical intervention and 767 (6.5%) with significant intracranial injuries. Fig 1 presents the flow chart detailing patient enrollment, while Table 1 presents the characteristics of the enrolled patients.

**Table 1:** Characteristics of the enrolled blunt head injury patients.

Trait	All	Intervention	Significant Injury
	(N = 11,770)	(N = 420)	(N = 767)
**Age (Years)**			
Median	50.0	59.4	59.0
IQR	29.0–71.6	33.9–77.8	35.4–78.7
Range	0.01–103.7	0.22–96.4	0.10–97.7
**Sex (N, %)**			
Male	7,217 (61.3%)	304 (72.4%)	541 (70.5%)
Female	4,526 (38.5%)	115 (27.4%)	224 (29.2%)
Unknown	27 (0.23%)	1 (0.24%)	2 (0.26%)
**Race (N, %)**			
Asian	631 (5.36%)	34 (8.10%)	71 (9.26%)
Black	1,241 (10.5%)	30 (7.14%)	51 (6.65%)
Middle Eastern	330 (2.80%)	11 (2.62%)	17 (2.22%)
Native American	7 (0.06%)	0 (0.0%)	0 (0.0%)
Other	649 (5.51%)	28 (6.67%)	51 (6.65%)
White	8,897 (75.6%)	317 (75.5%)	577 (75.2%)
Unknown	15 (0.13%)	0 (0.0%)	0 (0.0%)
**Ethnicity**			
Hispanic	2,013 (17.1%)	66 (15.7%)	131 (17.1%)
Non-Hispanic	9,742 (82.8%)	354 (84.3%)	636 (82.9%)
Unknown	15 (0.13%)	0 (0.0%)	0 (0.0%)

### Performance of the NEXUS instrument among the enrolled cohort

The NEXUS Head CT DI correctly assigned high-risk status to 420 of the 420 patients requiring neurosurgical intervention, yielding a sensitivity of 100.0% (95% CI: 99.1%–100.0%). The instrument correctly assigned low-risk status to 2,823 of 11,350 patients who did not require neurosurgical intervention to yield a specificity of 24.9% (95% CI: 24.1%–25.7%). None of the 2,823 patients assigned low-risk status required intervention, yielding a NPV of 100.0% (95% CI; 99.9%–100.0%).

The DI correctly assigned high-risk status to 759 of the 767 patients with significant intracranial injuries, yielding a sensitivity of 99.0% (95% CI: 98.0%–99.6%). The instrument assigned low-risk status to 2,815 of 11,003 patients who did not have significant injuries to yield a specificity of 25.6% (95% CI: 24.8%–26.4%). Significant injuries were absent in 2,815 of the 2,823 patients assigned low-risk status to yield a NPV of 99.7% (95% CI: 99.4%–99.9%). Box 2 describes the eight patients who sustained significant injuries and who were incorrectly assigned to low-risk status by the NEXUS Head CT DI.

**Box 2. Patients having significant injuries who were incorrectly classified as low risk by the National Emergency X-Radiography Utilization Study (NEXUS) Head computed tomographic (CT) decision instrument**

**Patient 1:** 63-year-old male with subarachnoid hemorrhage in the midbrain cisterns, left sylvan fissure, and a few frontal lobe sulci. Trace blood in the right sylvan fissure, right temporal lobe sulci, and in the right posterior parietal region.

**Patient 2:** 32-year-old male with mild to moderate pneumocephalus within the anterior cranial fossa ventral to the frontal lobes bilaterally and along the anterior interhemispheric fissure secondary to complex fracture across the inner and outer tables of the frontal sinuses. Trace subarachnoid hemorrhage visualized within the sulci over the frontal lobes. Bilateral subfrontal regional cerebral edema.

**Patient 3:** 10-year-old female with a comminuted right temporoparietal skull fracture with small associated epidural air and small subdural blood. Lateral and high right cerebral convexity sulcal effacement without midline shift or hydrocephalus.

**Patient 4**: 40-year-old female with acute subarachnoid hemorrhage in bilateral frontal, temporal, and parietal lobes, left greater than right.

**Patient 5**: 34-year-old male with small to moderate amount of subarachnoid hemorrhage in the left parietal sulci and right sylvan fissure. There is a small, 3-mm region of intraparenchymal bleeding in the right parietal lobe.

**Patient 6**: 61-year-old female with white matter edema to right and left frontal lobes, consistent with shear injury. No intracranial hemorrhage.

**Patient 7**: 62-year-old female with subarachnoid hemorrhage in the right and left parietal lobes. Small left subdural hematoma measuring 1.5 mm in thickness and increased space in the subdural region anterior to the right frontal lobe concerning for a subdural hygroma.

**Patient 8**: 45-year-old female with an open fracture of the left temporal bone associated with a small focus of pneumocephalus and a focal extra-axial hemorrhage within the left middle cranial fossa, measuring 5 cm x 1 cm x 3 cm and suspicious for epidural hematoma. Transverse fracture through the roof of the left posterior orbit and diastasis of the left temporal suture. Mild sulcal effacement of the sulci within the left middle cranial fossa.

Among the 420 patients requiring neurosurgical intervention, 1 or more of the high-risk criteria were documented as being definitely present in 416 patients. Four individuals, including 3 judged as unstable, were classified as high risk due to inability to assess all criteria. In no patients requiring intervention were all of the high-risk criteria documented as being absent. Table 2 presents the prevalence of individual criterion assignments for the total enrolled population, for patients with significant injuries, and those needing neurosurgical intervention. Abnormal level of alertness was present in 508 of the 767 (66.2%) patients with significant injuries, and 342 of the 420 (81.4%) patients requiring intervention, making it the most prevalent predictive criterion.

**Table 2:** Prevalence of predictor variables among the enrolled population.

Criterion	All	Significant Injury	Intervention
	(N = 11,770)	(N = 420)	(N = 767)
**Evidence of Skull fracture**			
No	11,187 (95.0%)	583 (76.0%)	293 (69.8%)
Yes	583 (4.95%)	184 (24.0%)	127 (30.2%)
**Scalp Hematoma**			
No	7,668 (65.1%)	339 (44.2%)	192 (45.7%)
Yes	4,102 (34.9%)	428 (55.8%)	228 (54.3%)
**Neurologic Deficit**			
No	9,708 (82.5%)	362 (47.2%)	124 (29.5%)
Yes	2,062 (17.5%)	405 (52.8%)	296 (70.5%)
**Abnormal Alertness**			
No	8,425 (71.6%)	259 (33.8%)	78 (18.6%)
Yes	3,345 (28.4%)	508 (66.2%)	342 (81.4%)
**Abnormal Behavior**			
No	9,017 (76.6%)	348 (45.4%)	123 (29.3%)
Yes	2,753 (23.4%)	419 (54.6%)	297 (70.7%)
**Persistent Vomiting**			
No	11,085 (94.2%)	645 (84.1%)	345 (82.1%)
Yes	685 (5.82%)	122 (15.9%)	75 (17.9%)
**Coagulopathy**			
No	8,048 (68.4%)	360 (46.9%)	148 (35.2%)
Positive	3,722 (31.6%)	407 (53.1%)	272 (64.8%)
**Age 65 or Greater**			
No	8,065 (68.5%)	436 (56.8%)	241 (57.4%)
Yes	3,705 (31.5%)	331 (43.2%)	179 (42.6%)

### Performance of the NEXUS and Canadian instruments

**Fig 2:**
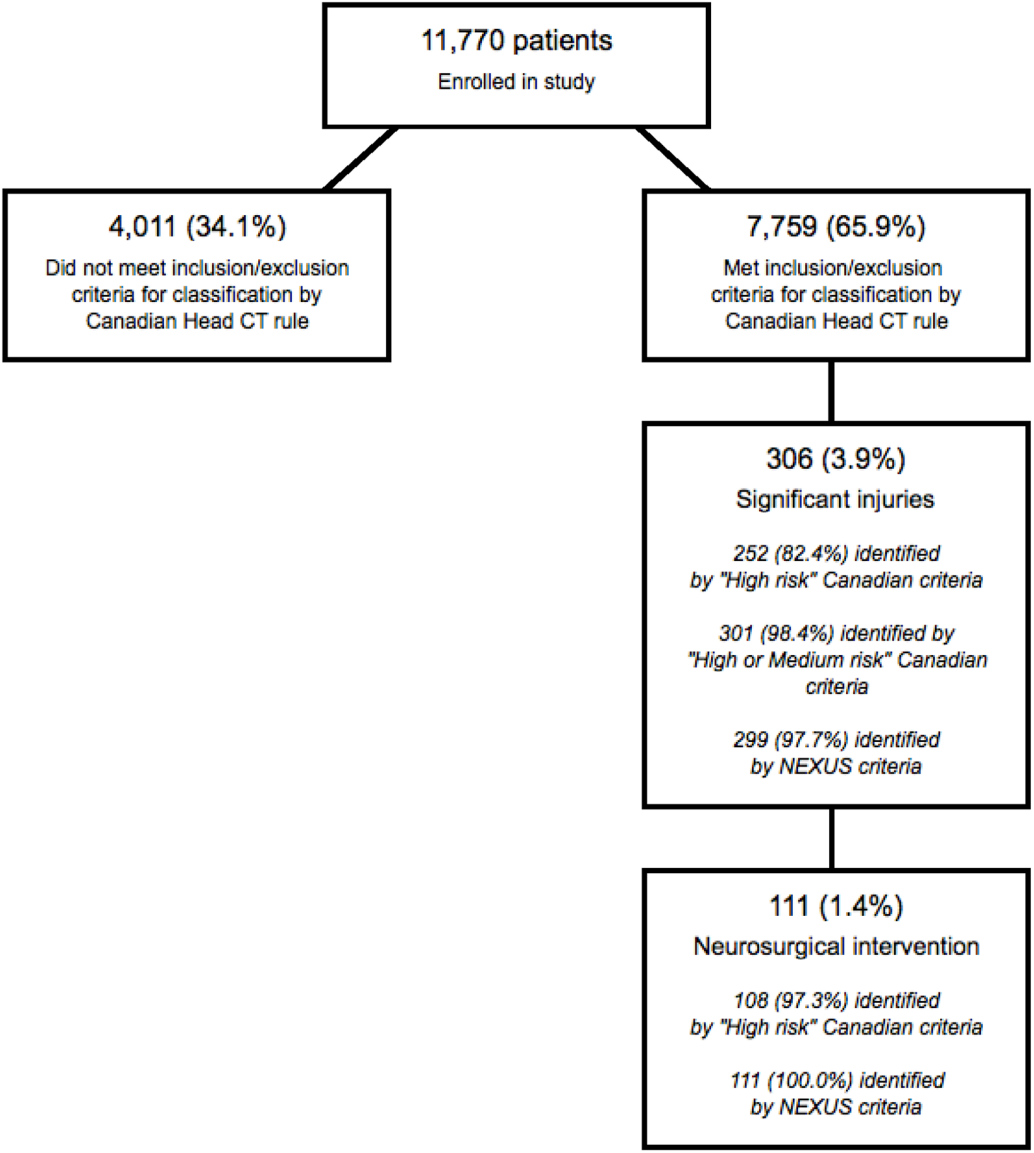
Identification of patients suitable for evaluation by the Canadian Head computed tomographic (CT) rule.

Our population included 7,759 (65.9%) patients who satisfied the inclusion and exclusion criteria for classification by the Canadian Head CT rule. This subpopulation included 111 patients (1.43%) who required neurosurgical intervention and 306 patients (3.94%) with significant intracranial injuries. Fig 2 presents the flow chart detailing the subclassification of the original cohort, while Table 3 presents the classification summaries, operator characteristics, and associated CIs for both the Canadian and NEXUS Head CT rules, as measured on this subcohort. The Canadian “high-risk” criteria for neurosurgical intervention identified 108 of 111 patients requiring neurosurgical intervention and 252 of 306 patients with significant injuries to yield a sensitivity of 97.3% (95% CI: 92.3%–99.4%) in detecting injuries requiring intervention and a sensitivity of 82.4% (95% CI: 75.2%–86.5%) in detecting significant injuries. Box 3 describes the 3 patients requiring neurosurgical intervention who were incorrectly assigned to low-risk status by the Canadian “high-risk” criteria. The NEXUS rule, when applied to this same cohort, identified all 111 patients requiring neurosurgical intervention to yield a sensitivity of 100% (95% CI: 96.7%–100.0%). The Canadian “medium-risk” criteria identified 301 of 306 patients with significant injuries (sensitivity = 98.4%; 95% CI: 96.2%–99.5%), while the NEXUS rule identified 299 of these patients (sensitivity = 97.7%; 95% CI: 95.3%–99.1%). The Canadian rule exhibited a specificity of 12.3% (95% CI: 11.6%–13.1%). In comparison, the NEXUS rule exhibited a specificity of 33.3% (95% CI: 32.3%–34.4%).

**Box 3. Patients incorrectly classified as low risk by the Canadian Head computed tomographic (CT) “high-risk” factors**

**Patient 1**

Male, age 16.5 years, walking as a pedestrian was struck by a car with initial brief loss of consciousness but no amnesia. A Glasgow Coma Scale (GCS) score of 15 was present on presentation and throughout emergency department course. The patient exhibited no evidence of skull fracture, and no vomiting, but exhibited National Emergency X-Radiography Utilization Study (NEXUS) criteria of scalp hematoma and abnormal level of alertness.

An initial CT revealed a 3-mm left-sided frontotemporal hematoma with associated temporal bone fracture and a right-sided 8 mm x 5 mm focus of subarachnoid blood. A follow-up 4 hour CT revealed interval increase in size of the left-sided epidural hematoma to 11 mm.

The patient underwent craniotomy and hematoma evacuation.

**Patient 2**

Male, age 53.1 years, fell 20 feet from scaffolding. The patient presented with a GCS score of 15 that was maintained thorough the emergency department stay, and had no amnesia, no evidence of skull fracture, and no vomiting but exhibited NEXUS criteria of scalp hematoma and abnormal level of alertness.

An initial CT revealed a linear occipital bone fracture across the jugular tubercle and foramen and right pterygoid plate, as well as bilateral subdural hematomas, parenchymal contusions, and subarachnoid bleeding with significant swelling in posterior fossa with compression of basal cisterns and loss of cerebral sulci.

The patient ultimately required intubation, ventriculostomy, and intracranial pressure (ICP) monitoring.

**Patient #3**

Male, age 59.4 years, sustained a ground-level fall with brief amnesia and a single episode of vomiting. The patient presented with and maintained a GCS score of 15 and exhibited no evidence of basal skull fracture and no further vomiting but exhibited NEXUS criterion of scalp hematoma.

An initial CT revealed a commuted and mildly displaced right frontoparietal skull fracture with underlying right convexity epidural hematoma 3-cm thick with mass effect and leftward shift of 6 mm, as well as subarachnoid hemorrhage along sulci of both convexities.

The patient underwent craniotomy and hematoma evacuation.

**Table 3:** Classification summary and operator characteristics of the Canadian and NEXUS decision instruments.*

**(a) Classification summary**				
**Decision** **Instrument**	**True Positives**	**False Negatives**	**True Negatives**	**False Positives**
**Neurosurgical intervention**				
**Canadian “high risk”**	108	3	4,498	3,150
**NEXUS**	111	0	2,490	5,158
**Clinically important injury**				
**Canadian “medium risk”**	301	5	917	6,536
**NEXUS**	299	7	2,483	4,970
**(b) Operator characteristics**				
**Decision** **Instrument**	**Sensitivity (95% Confidence interval)**	**Specificity (95% Confidence interval)**	**Positive Predictive Value** **(95% Confidence interval)**	**Negative Predictive Value** **(95% Confidence interval)**
**Neurosurgical intervention**				
**Canadian “high risk”**	97.3% (92.3–99.4)	58.8% (57.7–59.9)	3.3% (2.7–4.0)	99.9% (99.8–100.0)
**NEXUS**	100.0% (96.7–100.0)	32.6% (31.5–33.6)	2.1% (1.7–2.5)	100.0% (99.9–100.0)
**Clinically important injury**				
**Canadian “medium risk”**	98.4% (96.2–99.5)	12.3% (11.6–13.1)	4.4% (3.9–4.9)	98.5% (98.7–99.8)
**NEXUS**	97.7% (95.3–99.1)	33.3% (32.3–34.4)	5.7% (5.1–6.3)	99.7 (99.4–99.9)

* Population consists of the 7,759 patients meeting the inclusion and exclusion requirements of the Canadian Head CT rule.

CT, computed tomographic; NEXUS, National Emergency X-Radiography Utilization Study.

### Assessment of the potential for verification bias

Our substudy examining the potential for verification bias enrolled 368 patients, 356 of whom received no further imaging or treatment for their injuries. Twelve patients ultimately underwent cranial imaging during their recovery, including six who received head CT imaging, and six who received magnetic resonance head imaging. No injuries were found on these imaging studies, and none of the 368 patients required neurosurgical intervention, yielding a measured potential for verification bias of 0.0% (95% CI: 0.0%–1.0%). Our review of case and trauma logs failed to reveal any missed instances of significant intracranial injuries or injuries requiring neurosurgical intervention among head injury patients who were discharged without imaging on their initial presentation.

## Discussion

### Validation of the NEXUS Head CT DI

Our study demonstrates that the NEXUS Head CT DI is highly sensitive in detecting blunt trauma patients who harbor injuries that require neurosurgical intervention. Similarly, the high NPV provides reliable assurance that patients designated as “low risk” will not harbor these same injuries. The DI also exhibits extremely high sensitivity and NPV in detecting clinically significant injuries. Taken together, the precise measurements of sensitivity and NPV provide the validation needed to support the use of the tool in evaluating blunt head injury patients [15].

While the ability to detect and exclude injury is essential for the DI, its practical value lies in its ability to decrease imaging. Physicians in our study ordered imaging using whatever criteria they deemed appropriate and were specifically cautioned against using an unvalidated rule. The 11,770 patients selected for imaging included 2,815 (23.9%) patients who were ultimately classified as “low risk” by the tool. Application of the NEXUS Head CT DI would have allowed clinicians to safely omit imaging in these patients and suggests that the rule could significantly reduce CT imaging of blunt head injury patients. Taken together, these observations imply that the NEXUS Head CT DI is a reliable tool for identifying blunt head trauma patients at risk of important intracranial injuries while simultaneously offering the potential to reduce CT imaging.

### Implementing the NEXUS Head CT rule

In general, DIs that exhibit high sensitivity are best used in conjunction with clinical judgment. This approach is particularly valuable in implementing the NEXUS Head CT DI. Numerous studies and our assessment of verification bias all reveal that injury prevalence among unimaged patients is negligible [8,14,16,17]. Clinicians, using clinical judgment alone, are already very sensitive in identifying and imaging patients with important injuries. DIs are not needed to improve sensitivity, and applying DIs to patients that are not currently selected for imaging offers no advantage. Instead this practice is likely to unnecessarily increase imaging. The greatest value of the highly sensitive NEXUS DI arises from its ability to safely reduce imaging by improving the specificity of imaging decisions. Consequently, the NEXUS DI should only be applied after a clinical evaluation suggests that imaging is indicated; patients exhibiting all of the low-risk criteria may safely forego imaging.

### Assessment of the Canadian Head CT rule

A secondary goal of our study was to perform a more precise assessment of the Canadian Head CT rule. The initial development of the rule included only 44 patients requiring neurosurgical intervention. While the rule correctly classified all 44 patients as high risk, the corresponding lower CI for sensitivity was 92%, leaving questions about its validation [14,15]. This uncertainty has yet to be corrected by other studies [16,17].

In our study, among the subset of patients who met the inclusion and exclusion requirements, the Canadian Head CT “high-risk” criteria exhibited a sensitivity of 98.7% (with a lower confidence bound of 96.8%) in identifying patients requiring neurosurgical intervention. While these numbers are consistent with the original study, they raise concern regarding whether the rule has sufficient sensitivity in detecting injuries that require intervention. Review of the patients with missed injuries reveals that 2 of the 3 misses occurred in patients with epidural hematomas who experienced prolonged lucid intervals. In assessing the construct validity of the rule, it is apparent that the rule is vulnerable to such incidents. It is also important to note that 1 of the patients with missed injuries was not identified by the medium-risk factors of amnesia or dangerous mechanism and would have been classified as “low risk” by both the “high-risk” and “medium-risk” versions of the Canadian rule.

In our study, the Canadian “high-risk” criteria exhibited a specificity of 58.8% in identifying patients who do not have intracranial injuries requiring intervention. While this high specificity is advantageous, it comes at the cost of poor sensitivity in detecting significant injuries (sensitivity of 82.4%). Thus, while the high specificity is compelling, clinicians are unlikely to embrace the “high-risk” criteria in isolation because they are likely to miss nearly one-sixth of the patients having significant injuries. From a practical perspective, the reliable detection of injuries requires use of the full Canadian rule, including application of both the “high-risk” and “medium-risk” criteria.

With this in mind, it is worth noting that both the NEXUS and full Canadian rules exhibited similar high sensitivity and NPV in identifying patients with significant injuries. The primary difference between the rules lies in their potential utility. Because the NEXUS rule has no inclusion or exclusion criteria, it can be applied to all blunt head injury patients. In contrast, the Canadian rule has numerous restrictions and could not be applied to approximately one-third of our cohort (4,011 patients). Clinicians who employ the Canadian rule will have to find alternative means of assessing the need for imaging among such patients.

It is worth noting that we found lower specificity than that observed in the original Canadian study [14]. This difference reflects the large number of patients who were never imaged in the original Canadian cohort (1,043 of 3,121 patients), the majority of whom were classified as “low risk” by the instrument. The inclusion of such patients acts to increase the observed specificity of the Canadian rule, but limits an assessment of the ability of the rule to decrease imaging. We found that the full Canadian rule assigned “low-risk” status to 917 of the 7,987 patients who satisfied the inclusion and exclusion criteria. Assuming imaging could be omitted in all of these “low-risk” patients, the Canadian rule could potentially reduce imaging by 11.5%. In the same cohort, the NEXUS rule classified 2,571 patients as “low risk,” with the corresponding potential reduction in imaging of 32.2%, a nearly 3-fold reduction in imaging.

### Using DIs to guide CT head imaging

Clinical DIs such as the NEXUS and Canadian Head CT instruments are designed to serve as screening tools in the initial assessment of blunt head injury patients. Imaging may be safely omitted for patients who are classified as low risk, while imaging or another form of evaluation, such as prolonged observation, is appropriate for patients that do not meet low-risk classification [12].

Other tools, such as biomarkers, may also play a role in injury assessment, but the exact role these tools play in injury detection has yet to be determined [22,23]. For example, there are currently no reliable direct comparisons examining the performance of DIs and biomarkers to determine whether one means of injury assessment is superior to another. Further research will also be needed to examine the combined use of these tools and whether they are best used to enhance sensitivity, or whether they might play an important role in enhancing specificity and reducing imaging.

### Limitations

While we employed a naturalistic design in our study, screening performance results are likely to differ when applied in different environments. This is particularly true for our measured specificity and potential to reduce imaging. For example, centers that encounter a larger proportion of low-risk patients will likely find a higher proportion of patients that meet “low-risk” classification and are likely to find a higher specificity for the instrument. Hospitals that encounter a higher proportion of seriously injured patients may find fewer “low-risk” patients and observe a lower specificity. Similarly, clinicians differ from center to center, and while several hundred clinicians participated in our trial, their combined performance will likely differ from that of any individual or single center.

The criteria in our rule are also subject to interpretation. We tried to provide a pragmatic, “real world” exposure of these criteria to the clinicians participating in the study by providing them with definitions of the criteria but refraining from intense training in the assessment of each criterion. Most of our criteria are very straightforward, but interpretation of a few of the criteria and application of the rule may change as exposure to the rule changes. This may be particularly relevant to centers with cultural and language differences [24].

Because we employed neurosurgical intervention as a primary outcome in our study, our results are vulnerable to the decision process used in implementing these interventions. Other centers, with different neurosurgical support services, may find differing rates and levels of intervention. These differences could impact all of the measured operator characteristics.

Tracking outcomes for all blunt head injury patients for an extended period is not feasible. This implies that our assessment of the potential for verification bias is not exhaustive, and it is possible that a patient with serious intracranial lesions may have been discharged with unrecognized injuries. However, UCLA is the regional trauma and neurosurgical center, and it is likely that any patient with injuries missed on an initial evaluation would ultimately return for evaluation and intervention. Thus, while instances of missed injury may have occurred during the study, our failure to identify any such injuries indicates that they are extremely rare and well below significant thresholds.

## Conclusions

Our study validates the NEXUS Head CT DI and provides clinicians with a highly sensitive tool to guide selective imaging decisions in blunt head injury patients that may decrease head CT utilization in low-risk adult populations by up to 25% in comparison to standard practice that does not employ a DI.

## Supporting information

S1 STARD Checklist (DOCX)

S1 Protocol (DOCX)

## Abbreviations

CI: confidence interval
CT: computed tomographic
DI: decision instrument
ED: emergency department
GCS: Glasgow Coma Scale
ICP: intracranial pressure
NEXUS: National Emergency X-Radiography Utilization Study
NPV: negative predictive value
TBI: traumatic brain injury
