# Supplementary material for: Validation of the sensitivity of the National Emergency X-Radiography Utilization Study (NEXUS) Head computed tomographic (CT) decision instrument for selective imaging of blunt head injury patients: An observational study
Source: PLoS Med. 2017 Jul 11;14(7):e1002313. doi: 10.1371/journal.pmed.1002313 (PMC5507397; doi:10.1371/journal.pmed.1002313)
Supplement: S1 Protocol — (DOCX) [file pmed.1002313.s002.docx]

**Selective Imaging of Blunt Head Injury Patients: Validation of the NEXUS Head CT Decision Instrument**

Statement of Compliance

The study will be carried out in accordance with Good Clinical Practice (GCP) as required by the following:

- United States (US) Code of Federal Regulations (CFR) applicable to clinical studies (45 CFR Part 46; 21 CFR Part 50, 21 CFR Part 56, and 21 CFR Part 312)
- ICH E6; 62 Federal Register 25691 (1997)
- NIH Clinical Terms of Award

All key personnel (all individuals responsible for the design and conduct of this study) have completed Human Subjects Protection Training.

Table of Contents

Page

Statement of Compliance …………………………………………………………………. … 2

Table of Contents …………………………………………………………………………….. 3

List of Abbreviations ………………………………………………………………………….. 5

Key Roles ……………………………………………………………………………………… 6

Protocol Summary ……………………………………………………………………………. 7

1. Specific Aims …………………………………………………………………………… 7

2. Background and Significance ………………………………………………………… 8

3. Preliminary Studies …………………………………………………………………….. 17

3.1 - Previous work on selective criteria ………………………………………….. 18

3.2 - Sample size calculations …………………………………………………….. 20

4. Experimental Design and Methods ………………………………………………….. 21

4.1 Experimental Design …………………………………………………………… 21

4.2 Protocol Overview ……………………………………………………………… 22

4.3 Methods …………………………………………………………………………. 22

4.3.1 - Structure of the multicenter study and general administration ………. 22

4.3.2 - Data collection and x-ray ordering ……………………………………… 23

4.3.2.1 - Patient entry ………………………………………………………….. 23

4.3.2.2 - Assessing the potential for work-up bias ………………………….. 24

4.3.3 - Data collected …………………………………………………………….. 25

4.3.3.1 - Work-up bias assessment …………………………………………... 26

4.3.4 - Patient consent …………………………………………………………… 28

4.3.5 - Tomographic orders ……………………………………………………… 29

4.3.5.1 - Radiographic imaging ……………………………………………….. 30

4.3.5.1.1 - Radiographic evaluation ………………………………………… 30

4.3.5.1.2 - Injury diagnosis …………………………………………………... 30

4.3.5.2 - Radiographic request generation …………………………………... 31

4.3.6 - Database management ………………………………………………….. 31

4.3.7 - Data analysis ……………………………………………………………… 32

4.3.7.1 - Incomplete records …………………………………………………... 32

4.3.7.1.1 - Stable patients …………………………………………………… 32

4.3.7.1.2 - Unstable patients ………………………………………………… 33

4.3.7.2 - Mathematical calculations …………………………………………… 33

4.3.7.2.1 - Sensitivity, specificity and negative predictive value ………… 33

4.3.8 - Quality Assurance ………………………………………………………… 34

4.3.8.1 - Protection of survey data ……………………………………………. 34

4.3.8.2 - Protection of radiographic data integrity …………………………… 35

4.3.8.3 - Error processing and protection of data integrity …………………. 36

4.3.8.4 - Equipment failures …………………………………………………… 36

4.3.8.5 - Study site termination ………………………………………………... 37

4.3.9 - Difficulties and limitations ……………………………………………….. 37

4.3.9.1 - Sensitivity and negative predictive value ………………………….. 37

4.3.9.2 - Specificity ……………………………………………………………… 38

4.3.9.3 - External validity ………………………………………………………. 39

4.3.10 - Work schedule …………………………………………………………... 39

4.3.10.1 - Start-up period ………………………………………………………. 39

4.3.10.2 - Running schedule …………………………………………………... 39

4.3.10.3 - Duration of the study ……………………………………………….. 39

4.3.11 - Specific duties of study personnel …………………………………….. 40

5. Human Subjects ………………………………………………………………………... 41

5.1 - Risks to the subjects …………………………………………………………. 41

5.2 - Adequacy of protection against risk ………………………………………… 41

5.3 - Potential benefits of the proposed research to the subjects and others … 42

5.4 - Importance of knowledge to be gained ……………………………………... 42

5.5 - Inclusion of women …………………………………………………………… 42

5.6 - Inclusion of minorities ………………………………………………………… 43

5.7 - Inclusion of children …………………………………………………………... 43

5.8 - Data and safety monitoring plan …………………………………………...... 43

6. Literature Cited …………………………………………………………………………. 44

Appendix A – Participating Centers ………………………………………………………… 50

Appendix B – Blunt Head Trauma Questionnaire …………………………………………. 51

Appendix C – Detailed Description of Criteria Included in the NEXUS Head CT

Instrument ………………………………………………………………………………. 52

Appendix D – Scripted Survey Form for Long-Term Follow-Up of Patients Who Do

Not Undergo Tomographic Imaging During Their Initial Evaluation ………………. 53

Appendix E – Specific Duties of Study Personnel ………………………………………… 55

**List of Abbreviations**

| CA | California |
| --- | --- |
| CFR | Code of Federal Regulations |
| CAD | Coronary Artery Disease |
| CAT | Computed Axial Tomography |
| C.I. | Confidence Interval |
| COPD | Chronic Obstructive Pulmonary Disease |
| CT | Computed Tomography |
| ED | Emergency Department |
| F | F Distribution |
| FN | False Negative |
| FP | False Positive |
| GCP | Good Clinical Practice |
| GCS | Glasgow Coma Scale |
| HIPPA | Health Insurance Portability and Accountability Act of 1996 |
| ICI | Intracranial Injury |
| IRB | Institutional Review Board |
| ITP | Idiopathic Thrombocytopenia |
| LOC | Loss of Consciousness |
| K | Kappa statistic |
| mGray | miliGray |
| MRI | Magnetic Resonance Imaging |
| N | Number (typically refers to subjects) |
| NEXUS | National Emergency X-radiography Utilization Study |
| NIH | National Institutes of Health |
| PHS | Public Health Service |
| PI | Principal Investigator |
| P_L_ | Lower Confidence Limit |
| P_u_ | Upper Confidence Limit |
| QA | Quality Assurance |
| QC | Quality Control |
| TN | True Negative |
| TP | True Positive |
| UCLA | University of California, Los Angeles |
| UCSF | University of California, San Francisco |
| US | United States |
| X | Number (typically refers to subject related events) |

**Key Roles**

| Principal Investigator: | William R. Mower, MD, PhD  UCLA – Emergency Medicine Center  924 Westwood Blvd., Suite 300  Los Angeles, CA 90024  Phone number: 310-794-0582  Fax number: 310-794-0599  E-mail: wmower@ucla.edu |
| --- | --- |
| Site Investigators/  Institutions: | 1. William R. Mower, MD, PhD  UCLA – Emergency Medicine Center  924 Westwood Blvd., Suite 300  Los Angeles, CA 90024  Phone number: 310-794-0582  Fax number: 310-794-0599  E-mail: wmower@ucla.edu  2. Malkeet Gupta, MD  UCLA – Emergency Medicine Center  924 Westwood Blvd., Suite 300  Los Angeles, CA 90024  and  Antelope Valley Hospital Emergency Department  1600 W Avenue J  Lancaster, CA 93534  E-mail: [malkeet@gmail.com](mailto:malkeet@gmail.com)  3. Robert Rodriguez, MD  San Francisco General Hospital  Department of Emergency Medicine  UCSF School of Medicine  505 Parnassus Ave.  San Francisco, CA 94143  E-mail: Robert.rodriguez@ucsf.edu  4. Gregory Hendey, MD  UCSF Fresno, Community Regional Medical Center  Department of Emergency Medicine  2823 Fresno St.  Fresno, CA 93721  E-mail: ghendey@fresno.ucsf.edu |

**Protocol Summary**

**1. SPECIFIC AIMS**

Radiographic imaging can provide information that is crucial in managing victims of blunt trauma. However, liberal and indiscriminant imaging is expensive, can delay critical interventions, and unnecessarily exposes patients to ionizing radiation and its potential for lethal malignant transformation.

Considerable benefit could be realized by identifying clinical criteria that reliably classify the risk of specific injuries in blunt trauma patients. Patients identified as “no risk” by such criteria would not harbor specific forms of injury, and would receive no benefit from radiographic evaluation for such injuries. These patients could safely be spared the expense and radiation exposure associated with such imaging.

The purpose of this project is to identify and validate the use of clinical criteria to stratify the risk of specific injuries among blunt trauma patients. This proposal specifically addresses the use of such criteria in assessing patients for intracranial injuries, where our previous work suggests that a decision instrument, composed of a limited number of clinical criteria, can reliably identify blunt head injury victims who have “no risk” of significant brain injury, and therefore no need for computed head tomography. We estimate that nation-wide implementation of this decision instrument could safely reduce tomographic imaging of blunt head injury victims by more than 12%, and reduce annual radiographic charges by $90 million, while simultaneously expediting trauma care and decreasing radiation exposure and its associated risk of radiation-induced malignancy.

The specific aim of this proposal is reduce computed head tomography by validating the ability of a previously developed decision instrument to reliably identify blunt head injury victims who have no risk of intracranial injury requiring neurosurgical intervention, and who can subsequently be safely spared tomographic imaging. This goal will be accomplished by confirming the following two hypotheses:

I - Blunt trauma victims have no risk of intracranial injury requiring neurosurgical intervention, and hence no need for imaging, if they exhibit all of the following characteristics: 1 - No evidence trauma skull fracture; 2 - No scalp hematoma; 3 - No neurological deficits; 4 - Normal level of alertness; 5 - Normal behavior; 6 - No persistent vomiting; 7 - No coagulopathy; 8 - Age less than 65 years.

II - Implementation of a decision instrument that omits computed tomographic head imaging of blunt trauma patients who exhibit all of these criteria will reduce the number of computed head tomographs without missing any intracranial injuries that require neurosurgical intervention.

These hypotheses will be tested in a nationwide prospective multicenter study. The study will document the presence or absence of each criterion, as well as the presence or absence of intracranial injury in a cohort of blunt head injury victims. This information will be used to calculate the sensitivity, negative predictive value and specificity of the overall decision instrument. Validating the sensitivity of the decision instrument with high statistical confidence requires evaluations on 368 intracranial injury victims who require neurosurgical intervention, which in turn could require evaluations on as many as 31,000 blunt head injury patients.

Reductions in computed tomography will be determined by counting the number of head tomographs performed on “no risk” patients. Reductions in charges and radiation exposure will be determined by respectively summing radiographic charges and life-time decreases in radiation morbidity and mortality for all “no risk” cases.

**2. BACKGROUND AND SIGNIFICANCE**

It is estimated that approximately 1 million head injury victims undergo computed head tomography each year at an expense of $750 million. These tomograms revealed significant intracranial injuries in less than 60,000 patients, leaving the remaining 940,000 individuals (nearly 94%) with the expense and radiation exposure of a negative exam. The radiation exposure alone is expected to cause 46 cancers over the lifetime of these individuals [B-D].

The human and economic losses associated with over utilization of CT scanning, combined with medico-legal and quality assurance considerations, prompt the development of a decision instrument for selective computed head tomography of blunt head injury patients [1,2]. Practicing emergency physicians have identified the development of such an instrument as a high priority, and 97% of these clinicians indicated they would be willing to consider implementing a well-validated head CT decision instrument [3].

The current liberal use of computed tomography in blunt head trauma stems from several reports claiming significant intracranial injuries in patients sustaining “minor” head trauma [4-21]. Fear of missing these injuries, and their potential to produce severe neurologic disability or even death, has led some authors to advocate computed tomographic imaging in virtually all blunt head injury victims [9,15,18-20]. This strategy generates large numbers of negative CT scans for each injury detected [22-25]. Furthermore, many of the injuries detected by tomography do not require medical or surgical intervention [22,25,26].

Careful evaluation of the reported cases of “occult” intracranial injuries raises questions about the existence of such an entity. Many of the asymptomatic injury reports actually represent cases of poor documentation or inadequate evaluation. Review of the published head injury literature fails to reveal a single case of truly “occult” injury. Many of the studies documenting “occult” intracranial injuries in blunt trauma victims suffer from poor definitions of “mild” and “minor” head injuries. The study of “alert” patients presented by Fisher [4], includes individuals who were confused, disoriented and exhibited cranial nerve deficits. Jones reviewed records of patients admitted for neurologic observation. These individuals were assumed to be able to “talk and obey commands,” but details of neurologic evaluations are neither discussed nor presented [5]. Individuals having Glasgow coma scores of 13 or better were defined as having minor head injuries in Rimel’s studies, and individuals with a Glasgow comma scale of 15 were considered to be normal [6,7]. The Glasgow coma scale (GCS) was developed in 1974 as an aid in predicting long-term prognosis six hours post head injury [27]. Since this time, GCS scoring has been used as a mini-neurological exam in many settings. Unfortunately, GCS suffers as a screening tool. Patients may have GCS scores of 15 (perfect), despite focal neurologic abnormalities [11,21,27], while patients having GCS scores of less than 15, by definition, have neurologic impairment [27].

The cases in Marshall’s study [8], taken from the National Traumatic Coma Data Bank, and Rockswold’s study [9], all had admission Glasgow coma scores of 8 or less. Although each of these individuals “uttered at least some comprehensible speech” or “spoke intelligible words,” none had normal neurologic examinations [8,9]. Leclercq’s report on intracerebral hematomas without neurologic deficits, provides no details on either the source of the data or specific neurologic signs or symptoms. Of note, four of his eleven patients had documented amnesia or seizures associated with their head injury [10]. Dacey, using Rimel’s definition of minor head injury, found that the Glasgow coma scale was not reliable in excluding intracranial injury. Additional neurologic evaluations were not performed in this study [11]. All eight patients having intracranial injuries in Feuerman’s study, had neurologic impairments documented in their medical records [12]. Rivara’s data, taken from chart review, fails to identify clinical findings that reliably predict CT abnormalities. Unfortunately, the study fails to document the extent of missing chart review data. In addition, many of the CT abnormalities are of no clinical significance and are unrelated to intracranial injuries [13].

Klauber documented unexpected mortality in “low risk” patients. Unfortunately, risk classification was based entirely on the motor portion of the GCS in conjunction with pupillary reactivity. Additional neurologic testing, including the remainder of the GCS, was not used in risk classification [14]. In Stein’s studies, data from medical record review indicates that GCS alone is inadequate in reliably predicting CT abnormality or intracranial injury. Stein’s studies do not examine additional neurologic signs, nor do they document the extent of missing data. Thus it is impossible to know whether any of the reported injuries were truly “occult” [16,19,20]. The article by Ashkenazi presents six cases of delayed epidural hematoma. Despite the title of the article, all cases clearly exhibited neurologic impairment on presentation [15]. Livingston assessed patients using GCS and history of loss of consciousness. These two factors were unreliable in excluding intracranial injury. Evaluation of each patient was limited, and due to insufficient data collection, it is impossible to determine whether intracranial injuries occurred in any asymptomatic patient [17]. Harad’s chart review data suggests that GCS is unreliable in predicting CT abnormalities. Unfortunately, the study mixes both blunt and penetrating head injury cases, and is marred by missing data. Also, many of the documented CT abnormalities are of no clinical significance [18]. Finally, Lobato’s data, describing severely head injured patients, documents deterioration in individuals who initially “uttered at least some comprehensible speech”. Patients were assessed using only the verbal portion of the GCS; additional neurologic impairments were not documented [21].

There are currently no reliable reports of truly occult intracranial injuries in blunt head trauma victims. Studies documenting occult intracranial injuries suffer from poor definitions of significant injuries and typically include patients who have significant neurologic impairment, such as abnormal Glasgow coma scores [6,7,8,11,12,15,16,17,18,19,21], or fail to document neurologic function [4,5,9,10,11,13,14,17,21]. Injuries invariably occur in individuals who have identifiable findings on history or physical examination. This observation has led several investigators to suggest that clinical criteria could be used to selectively screen head injury victims for intracranial injuries [22,23,25,28-30].

Several investigators have attempted to use single findings to reliably identify patients at risk of intracranial injuries. The presence or absence of skull fracture has repeatedly been assessed as a risk factor for intracranial injury [31,32,33,34,35]. Thornbury found that skull fractures increased the risk of intracranial sequelae, however, all significant intracranial injuries in skull fracture victims could have been identified using clinical criteria. Detecting skull fractures did not improve the identification of intracranial injury victims [31]. Master’s study demonstrated that most patients with intracranial injuries do not have skull fractures and that most patients with fracture do not have significant intracranial sequelae [32]. Servadei studied the combined reliability of skull fracture and GCS assessment in excluding intracranial injuries. He found that the absence of skull fracture did not reliably exclude intracranial injury in patients having GCS of 14 or better [33,34]. A chart review by Mendelow confirms that skull fracture is unreliable in excluding intracranial injury in patients who “talk sensibly (verbal score 5 on the Glasgow coma scale)” [35]. Thus, skull fracture identification alone does not improve the detection of significant intracranial injuries, and assessment strategies aimed at diagnosing skull fractures have largely been abandoned in the US [31]. Furthermore, although injuries such as non-displaced linear skull fractures may coexist with other serious intracranial injuries, linear skull fractures themselves require no therapy [8,11,12,22,32].

Data from chart review has failed to identify criteria that reliably exclude intracranial injury. In a review of 183 intracranial hematoma patients, Miller found that GCS of 15, no headache, no vomiting, absence of neurological deficit, and absence of signs of basilar skull fracture were unreliable in excluding intracranial injury. Miller, however, analyzed each factor individually and did not evaluate combined criteria [36]. In an abstract, Shah asserts that GCS of 15, non-focal neurologic exam, absence of seizure, and absence of skull fracture were unreliable in excluding injury in 30 patients having intracranial pathology. It is unclear how these patients were identified and whether each patient lacked all criteria. The authors also fail describe the extent of neurologic examination or the source of their data [37]. Moran evaluated the reliability of GCS, history of loss of consciousness (LOC), and focal neurologic deficit in 200 head injured patients. Only two patients had surgically significant injuries, and both of these patients experienced LOC [28]. Taheri reviewed 310 head injuries in patients presenting with GCS of 15. Five victims had intracranial injuries, and all of these individuals had either unspecified neurologic deficits or basilar skull fractures [38].

In two studies, Reinus found that neurologic exam, intoxication, amnesia, and focal neurologic deficit failed to predict all abnormal tomograms in blunt head injury victims. The studies are limited in that they fail to document the extent of missing chart review data, and many of the CT abnormalities are of no clinical significance [39,40]. Based on chart review, Mohanty concluded that patients having GCS of at least 13, and no evidence of basilar skull fracture, received no benefit from tomographic scanning. Unfortunately, only 12 patients had abnormal CT scans, and the majority of these were clinically insignificant [41]. Borczuk evaluated 25 different findings from 1,448 case records and found that cranial soft-tissue injury, focal neurologic deficit, basilar skull fracture, and age over 60 identified all 11 injured patients. The study is limited by the small number of injured patients; it is impossible to reliably validate 25 variables using a sample size of 11. The authors appropriately recommend a larger prospective study [24]. Finally, Cullota reviewed 3,370 case records of patients experiencing LOC and presenting with GCS of 13 to 15. Neither abnormal GCS nor LOC reliably identified all intracranial injuries among this population [26].

Because of problems inherent in review studies, there is a need for a large prospective evaluation of selective criteria for detecting intracranial injuries. This is true for several reasons. First, much of the gathered information is incomplete and may be subject to biased interpretation. In our own recent review we found important data elements were frequently missing. It was impossible to determine whether specific findings were not documented, had not been evaluated, or were truly absent [42]. The head injury studies reviewed above uniformly failed to reveal the extent of missing data or how undocumented findings were analyzed. Furthermore, most did not document criteria used to select patients for CT scanning. Therefore the results may represent significant selection bias. Few studies examined neurological testing beyond GCS scoring [24,28,37-40], and only two authors documented specific neurologic findings [24,39]. Finally, in many of the older papers, skull films were used to select a high-risk population [31,33-35], a practice now rarely performed in the US [32].

The review papers do suggest that 5% to 18% of CT scans will be abnormal in head injury cases [11,12,16,18,24,30], while the incidence of significant intracranial injury varies from 0.1% [24] to 5% [16]. These differences can be explained in part by differences in the criteria for obtaining CT scans and to changes in operative indications over the last ten years.

The issue of “occult” injury is further complicated by imprecise definitions of “significant injury.” Many authors count any abnormality found on CT scan, whether clinically significant or not [13,24,28,39-41]. It is frequently impossible to distinguish insignificant findings from more serious injuries on the basis of data presented in papers. Classically, significant CT findings include any air or blood in the cranial vault [11,22,33,34], contusion [22,33,34] traumatic infarction [11], and complex or depressed skull fractures [11,22]. While some cases of these injuries are now treated non-operatively, all are considered indications for admission and observation [43-46].

Several small prospective studies have examined selective criteria in evaluating blunt head trauma [22,23,25,29,47]. Miller scanned all patients presenting with LOC or amnesia. Data forms were prospectively completed by physicians prior to CT imaging. However, the study did not examine neurologic function beyond GCS scoring. Abnormal CTs occurred in 84 of 1,382 patients (6.1%). Most of the abnormal findings were clinically insignificant. Only three patients required medical or surgical intervention; two patients required elevation of depressed skull fractures, both of which were clinically obvious, and one patient had a partial lobectomy on day 13 of their hospital stay [25]. Miller’s study suggests that selective criteria may be useful, but the small number of injuries makes it impossible to make reliable conclusions.

Schynoll studied 264 patients who had a history of blunt head trauma within two weeks prior to their presentation. Participating clinicians evaluated patients and completed a 51-point data form prior to obtaining computed tomography. The study collected data from both history and physical examination. Fifteen patients had abnormal CT scans and three required surgical intervention. All patients with a positive CT scan had at least one of the defined high-risk criteria [23]. The study suggests that, given enough criteria, it may be possible to identify all patients with intracranial injury. However, it is impossible to make statistically reliable conclusions with such a small sample size (three significant injuries). In addition, it is also unclear whether 51 criteria would be clinically useful. Evaluating 51 items would be difficult in routine practice and would probably be ineffective in reducing the need for CT.

Mikhail enrolled head injury victims who underwent CT and had GCS of 13 or better. Thirty-one variables were measured on each victim. Surgically correctable lesions were found in 3 of 35 enrollees, all of whom had at least one of the study variables. Logistic regression revealed headache and age to be statistically related to risk of intracranial injury [47]. This study, like Schynoll’s, suffers from inadequate small sample size and too many study variables.

In Jeret’s study, neurologists collected 21 history and examination elements on 712 patients with mild head injury. Sixty-seven patients (9.4%) had significant CT abnormalities (not including linear skull fractures or soft tissue injury), and two patients required surgical intervention. No single element or combination of elements predicted all CT abnormalities. However both surgical patients exhibited at least one of the elements [22]. Again, conclusions about selective criteria are impossible due to sparse sample size.

In a two-phase observational study, Madden attempted to isolate a set of selective criteria capable of detecting all significant intracranial injuries, while simultaneously reducing the overall need for computed tomography. In the first derivation phase, 51 clinical variables were prospectively evaluated on patients undergoing head CT. Regression analysis, performed on subsets of these variables, was used to identify the combination of criteria that correlated highest with the presence of injury. Ten variables were subsequently identified as potential low risk criteria. In phase two of the study, these low risk criteria were applied to a second cohort of 273 patients. Five patients had false negative clinical screens, defined as the need for admission despite low risk stratification. Of these five, three had linear skull fractures, one had a small epidural and one had a small amount of subarachnoid blood. No patients with low risk criteria required operative intervention [29].

More recently, Haydel proposed guidelines for selective imaging of patients who had sustained LOC [62]. Unfortunately, the validation phase of the study, conducted at a single institution, contained only 909 total head injury victims, and contained too few patients with significant intracranial injuries to allow sensitivity to be assessed with an acceptably narrow 95% confidence interval. Furthermore, the study used any acute CT abnormality as a primary outcome measure, ignoring the fact that many of these abnormalities are clinically insignificant. Finally, the rule has low specificity and it is unclear whether it is capable of reducing the overall use of tomographic imaging [63]. One of the criteria in particular, injury above the clavicles, was only assessed in patients already selected for tomographic imaging. It is likely that this criterion would lead to an increase in the overall use of head CT imaging if it were used to guide imaging of all blunt trauma patients. These limitations, have severely hampered the acceptance of these guidelines among emergency medicine practitioners [64].

In a Canadian study, Stiell, *et al*, employed more rigorous methodology in developing a guideline for imaging of adult blunt trauma patients who have sustained head injuries. This guideline recommends imaging for patients who exhibit any of the following five high risk factors: age 65 years or greater, persistent abnormal GCS scores (<15 for more than 2 hours), suspected open skull fracture, any sign of basal skull fracture, or persistent vomiting (two or more episodes). The criteria in this derivation study identified all cases requiring neurosurgical intervention, but the 95% confidence interval associated with this sensitivity were relatively wide (92 – 100%). This decision instrument also identifies “medium risk factors” (dangerous mechanism of injury, amnesia before impact > 30 minutes) that can be used to identify clinically significant injuries with 98.4% sensitivity (95% CI: 96 – 99%). The authors indicate that their decision rule would have advised imaging in only 54% of their blunt head injury population. However, many of the enrolled patients were followed clinically and never received imaging. The authors do not reveal how many of these patients would require imaging under the decision instrument, nor whether the instrument would actually decrease scanning among patients currently selected for imaging [63].

While the findings from this study are encouraging, the confidence intervals on the overall sensitivity, and the sensitivity for neurosurgically important lesions are excessively wide. It is likely that application of the decision instrument could readily miss up to 4% of significant intracranial lesions, and up to 8% of neurosurgically important injuries. The decision instrument itself is not applicable to all head injury patients, and contains a substantial list of exclusions, including “minimal head injury” (i.e. no loss of consciousness, amnesia, or disorientation), depressed skull fractures, acute focal neurological deficit, unstable vital signs, seizure, coagulopathy, GCS < 13 on presentation, pregnancy, and age less than 16 years [63]. Furthermore, the study was conducted at ten Canadian academic centers, which limits its external validity and applicability to community hospitals and U.S. practice standards. Finally, the recursive-partitioning scheme used to develop the rule provides an optimal fit only for the data gathered in the study and does not guarantee that the rule will retain its performance when applied to new patients [63]. Before this rule can be used in clinical practice, its performance must be validated in a new cohort of blunt head injury victims. This separate validation study must be conducted in a variety of medical environments and must enroll the large number of cases needed to precisely define the confidence intervals associated its performance.

Our work represents the most recent and rigorous attempt to develop a decision instrument for tomographic imaging of blunt head injury patients. By applying recursive partitioning to a prospective cohort of 13,728 blunt head injury victims undergoing tomographic imaging, we found that eight criteria were independently and highly associated with intracranial injury. These criteria included evidence of significant skull fracture, scalp hematoma, neurologic deficit, altered level of alertness, abnormal behavior, persistent vomiting, coagulopathy and age greater than 65 years. These criteria identified clinically important intracranial injuries with 98.3% sensitivity (95% CI: 97.2 – 99.0%), and 99.1% negative predictive value (95% CI: 98.5 0 99.5%). A decision instrument composed of these criteria would have identified 901 of 917 clinically important intracranial injuries. Furthermore, none of the 16 missed injuries required neurosurgical intervention. Application of the decision instrument would have decreased tomographic imaging by 12.9% [65]. As mentioned previously, recursive partitioning alone cannot guarantee that the identified criteria will exhibit acceptable performance when applied to a new cohort. Consequently, the performance of a decision instrument based on these criteria needs to be confirmed by a separate validation study.

Our work also suggests that the relatively small sample size used in the Canadian study may have led to overly optimistic results. In particular, the five “high risk” factors identified by the Canadian study (age 65 years or greater, persistent abnormal GCS scores, suspected open skull fracture, any sign of basal skull fracture, or persistent vomiting), were found in only 869 of the 917 intracranial injury cases identified in our study. This equates to a sensitivity of 94.8% in detecting significant intracranial injury. Fourteen of the missed injury cases required neurosurgical intervention, and provide concrete evidence that the Canadian “high risk” criteria are not 100% sensitive in detecting these important lesions. Errors in the Canadian instrument likely result from the fact that a GCS of 15 is not equivalent to a normal neurological exam. Patients can exhibit subtle neurologic impairments such as altered level of alertness and behavioral abnormalities, and still attain a “normal” GCS score of 15. It is also likely that the additional criteria identified as being important in our work (scalp hematoma and abnormal behavior), are important subtle indicators of intracranial injury that are essential in producing a decision instrument with high sensitivity. Because we did not collect specific information on traumatic amnesia and injury mechanism, we are unable to assess the overall sensitivity of the Canadian instrument, and whether it would decrease imaging among our cohort.

Because of the previously mentioned issues, there is a need for prospective validation of the proposed decision instruments for selective tomographic imaging of blunt trauma victims. We therefore propose an observational study that will document the clinical presentation of blunt head injury victims prior to computed tomographic imaging and combine this observational data with CT results and need for neurosurgical intervention to determine whether combinations of clinical criteria can reliably detect all patients at risk of significant intracranial injuries, reliably exclude injury in low risk patients, and reduce the overall number of CT evaluations in blunt head injury victims.

In particular, we propose evaluating the performance of a decision instrument composed of the following criteria: 1 - evidence of significant skull fracture, 2 - scalp hematoma, 3 - neurologic deficit, 4 - altered level of alertness, 5 - abnormal behavior, 6 - persistent vomiting, 7 - coagulopathy, and 8 - age greater than 65 years. Furthermore, while it is not the primary goal of this study, we propose a concurrent evaluation of the Canadian decision instrument composed of the following criteria: 1 - age 65 years or greater, 2 - persistent abnormal GCS scores, 3 - suspected open skull fracture, 4 - any sign of basal skull fracture, 5 - persistent vomiting, 6 - dangerous mechanism of injury, 7 - amnesia before impact > 30 minutes. This additional evaluation entails minimal additional burden for the study, and offers the potential to not only validate decision instruments for tomographic imaging, but also enable us to identify the most optimal strategy in terms of reduced CT usage.

Statistically reliable criteria must exhibit a sensitivity quotient of roughly 368/368 to be 95% certain that the true sensitivity is greater than 99.0% (i.e. to validate the sensitivity at the 99.0% confidence level with 95% certainty, the decision instrument would need to identify 368 consecutive intracranial injuries requiring neurosurgical intervention without any misses). The prevalence of neurosurgically important intracranial injuries among blunt trauma victims is between 1.2% and 1.4% [64, 65], which means that approximately 31,000 patients will be needed to guarantee a sample size large enough to provide the necessary statistical significance [48]. Thus, nearly 31,000 blunt head injury victims will be needed to validate the decision instrument for selective tomographic imaging.

We estimate that the annual nationwide reduction in charges resulting from applying selective criteria to blunt head trauma victims to be at least $90 million. We base this estimate on data from prior studies showing that in the US we obtain over 1 million CT scans on patients with mild head injury per year. In Borczuk’s paper [24], 1,448 patients had CT scans performed for minor head injury over a 15-month period. This represents approximately 1,159 CT scans per year in an emergency department with an annual census of 65,000. There are nearly 100 million emergency department visits per year in the US [51]. Extrapolating from Borczuk’s experience suggests that approximately 1.79 million CT scans are performed nation-wide each year. Madden’s data confirms these figures [29]; 813 patients had CT scans performed for minor head injuries over an 18 month period. This represents approximately 542 scans per year in an emergency department with an annual census of 32,000. Extrapolation suggests that approximately 1.7 million CT scans are performed annually in the Nation’s emergency departments. Assuming these institutions perform significantly more CT scans than other US hospitals, it is reasonable to conservatively estimate that one million CT scans are performed for head injury in the US each year. Total charges for a CT scan (without contrast) at our institution are $750. Assuming that most hospitals assess similar fees implies that the annual charges for radiographic screening for intracranial injury in blunt trauma victims exceeds $750 million a year. Our previous work revealed that application of the decision instrument for selective tomographic imaging could reduce imaging by 12.9%, suggesting that savings of nearly $90 million could be realized annually through the general use of the instrument.

The monetary benefits derived from such a policy must be weighed against any excess morbidity incurred by patients who suffer neurologic consequences because of a failure to diagnose occult intracranial injury. There would be no cost if the negative predictive value of the low-yield criteria was 100% for lesions capable of producing neurologic injury. Alternatively, the utility of selective criteria would decrease with any decrease in the negative predictive value. If selective criteria had a true negative predictive value of 99.0%, then 10,000 blunt head injury victims would be inappropriately classified each year. As many as 1.4% of these cases could harbor intracranial injuries requiring neurosurgical intervention [65], therefore 140 intracranial injuries could be missed each year. Miller estimates that 23% of missed intracranial injuries produce neurologic sequelae [36], much lower numbers have been suggested in the British literature [52]. Taking the higher figure, selective criteria could annually miss up to 32 potentially preventable adverse outcomes. Severe disability or death associated with head injury results in lifetime costs of approximately $350,000 (including medical care, rehabilitation and lost wages) [53]. Assuming a worst-case scenario, where each missed injury results in death or severe disability, it is possible that $12 million would be needed to provide lifetime care for 32 such injuries. Thus, even with 99.0% negative predictive value, there could be annual dollar savings of over $75 million.

Such analyses do not account for the benefits of reduced x-ray exposure that would result from such a policy. A standard head CT without contrast exposes thyroid tissues to an effective radiation dose equivalent of 0.5 mGray [54]. The estimated number of excess thyroid cancer cases among exposed individuals over the age of five years is 10/10,000 person-year-Gray (excess cancer cases among persons exposed under age five is over 30/10,000 person-year-Grays) [55]. The average radiated trauma victim is approximately 35 years of age and can be expected to live for an additional 43 years [22,25,47,56]. Thus, in total, these figures predict 22 excess thyroid cancers over the lifetime of the one million trauma victims radiated each year. These results are similar to the cancer estimates derived by Huda [57]. In addition, other radiated head and neck tissues contribute to the overall cancer risk and may be expected to develop an additional 24 malignancies [57]. Reducing CT scans by 10% will directly reduce the excess cancer morbidity and mortality by 10%. Thus reducing head CT scanning by 10% will prevent malignancies in 5 individuals. This would in part offset the morbidity of the 32 potentially correctable intracranial injuries that might, in the worst-case scenario, fail to be identified by selective criteria.

A reliable decision instrument would not only produce significant health-care savings, and improve patient care, but could also be important in reducing practice variation and defining practice standards [63]. Emergency physicians indicate they are willing to adopt a reliable instrument for CT head imaging [3], and studies of decision instruments for plain radiography reveal substantial reductions in imaging [66, 67]. Alternatively, the failure to develop a reliable guideline for CT head imaging will likely result in increased imaging in the future [63]. Several recent publications strongly suggest that all patients with head injury, including those with minor head injury, undergo imaging [17, 68-70]. This practice could increase imaging among minor head injury victims by more than 300%, and make the validation of a decision instrument for tomographic head imaging a priority among emergency physicians [3, 63].

It is important to note that the primary goal of this study is to validate a clinical decision instrument that will identify patients who are unlikely to have findings on tomographic scanning. This study is not designed to determine the ultimate management of these patients. However, current information suggests that it is safe to discharge patients who have negative tomographic scans, provided they have a normal neurologic exam, do not have coagulopathy, and are discharged into the care of a reliable individual who can procure medical assistance in the event of neurological deterioration [49, 65]. Because the decision rule developed in this study will reliably identify blunt head injury victims who have no evidence of intracranial injury (as determined by initial tomography), it is likely that physicians will discharge many patients found to be free of the risk criteria. However, it is beyond the scope of this project to determine the final disposition of patients who have initially normal CT images.

**3. PRELIMINARY STUDIES**

The concept of using a limited set of criteria to identify patients at risk of serious injury is not new to this study. In this section we will present our previous experience in developing selective criteria for computed head tomography in blunt trauma. The methodology used in our previous work is similar to the methodology developed for this proposal. This section also describes our candidate criteria and contains our sample size calculations.

**3.1 - Previous work on selective criteria.**

Phase I - Interrater Reliability

*Inter-rater Reliability of Criteria Used in Assessing Blunt Head Injury Patients for Intracranial Injuries*

Judd E. Hollander, MD, Steven Go, MD, Doug W. Lowery, MD, Allan B. Wolfson, MD, Charles V. Pollack MD, Mel Herbert, MBBS, William R. Mower, MD, PhD, Jerome Hoffman, MD, MA.

*Acad Emerg Med* 2003;10:830-5 [71].

Purpose: To determine the inter-rater variability of criteria used to evaluate blunt head injury victims for evidence of intracranial injury.

Methods: Physicians from 21 separate emergency departments performed paired evaluations of patients undergoing computed tomographic head imaging. Each physician independently determined whether each patient had any of the following characteristics: 1) eyes open spontaneously, 2) oriented, 3) follows commands, 4) seizure following trauma, 5) loss of consciousness, 6) prolonged loss of consciousness, 7) severe or progressive headache, 8) coagulopathy, 9) abnormal behavior, 10) abnormal level of alertness, 11) evidence of significant skull fracture, 12) persistent vomiting, 13) evidence of intoxication, 14) motor deficit, 15) gait abnormality, 16) abnormal cerebellar function, 17) cranial nerve abnormality, 18) inability to read or write, 19) presence of scalp hematoma, 20) neurological deficit. Responses were compared using the kappa statistic on a sample size of 3,951 patients, with a lower confidence value of 0.50 or greater interpreted to represent substantial inter-rater agreement.

Results: The physician evaluations exhibited the following kappa values: 1) eyes open spontaneously [Κ = 0.68], 2) oriented [Κ = 0.77], 3) follows commands [Κ = 0.76], 4) seizure following trauma [Κ = 0.42], 5) loss of consciousness [Κ = 0.82], 6) prolonged loss of consciousness [Κ = 0.71], 7) severe or progressive headache [Κ = 0.69], 8) coagulopathy [Κ = 0.73], 9) abnormal behavior [Κ = 0.64], 10) abnormal level of alertness [Κ = 0.66], 11) evidence of significant skull fracture [Κ = 0.62], 12) persistent vomiting [Κ = 0.66], 13) evidence of intoxication [Κ = 0.84], 14) motor deficit [Κ = 0.72], 15) gait abnormality [Κ = 0.77], 16) abnormal cerebellar function [Κ = 0.58], 17) cranial nerve abnormality [Κ = 0.69], 18) inability to read or write [Κ = 0.70], 19) presence of scalp hematomas [Κ = 0.68] 20) neurological deficit [Κ = 0.79].

Conclusions: We found substantial agreement between clinicians in their assessments of all criteria except the presence of seizure following injury.

Phase II - Derivation of Selective Criteria

*Developing a Decision Instrument to Guide Computed Tomographic Imaging of Blunt Head Injury Patients*

William R. Mower, MD, PhD, Jerome R. Hoffman, MD, MA, Mel Herbert, MBBS, Allan B. Wolfson, MD, Charles V. Pollack, Jr, MD, Michael I. Zucker, MD: NEXUS II Investigators.

*J Trauma* 2005;59:954-9 [74].

Purpose: To determine if a limited number of clinical criteria can reliably identify significant intracranial injuries in blunt trauma victims without missing any significant cases.

Methods: Clinicians assessed the presence or absence of candidate criteria on 13,728 consecutive blunt trauma patients for whom computed head tomography was ordered. Clinicians completed their assessments prior to obtaining imaging results. CT imaging results were reviewed to determine which patients had intracranial injuries. Recursive partitioning was used to identify which candidate criteria reliably predicted injury.

Results: The study enrolled 13,728 patients, including 917 with clinically important intracranial injuries. Recursive partitioning identified eight criteria that were highly associated with intra-cranial injury. These criteria include: 1 - evidence of significant skull fracture, 2 - scalp hematoma 3 - neurologic deficit, 4 - altered level of alertness, 5 - abnormal behavior 6 - persistent vomiting, 7 - coagulopathy, 8 - age 65 years or greater. This rule exhibits 98.3% sensitivity (C.I.: 97.2 – 99.0), a 99.1% negative predictive value (C.I.: 98.5 – 99.5), and a specificity of 13.7% (C.I.: 13.1 – 14.3). Implementation of this rule would have identified all but 16 of 917 patients having clinically important ICI, and would have correctly classified 1,752 (12.9%) patients as “low risk.” Follow-up evaluations, performed on 1,266 patients who did not undergo computed tomography during their initial presentations, reveal that a small minority of these patients ultimately receive cranial imaging. However, none of the follow-up cases required hospitalization or neurosurgical intervention, and there were no cases of missed intracranial injuries or death among this cohort.

Conclusions: We found that a limited number of clinical criteria can reliably identify significant intracranial injuries in blunt trauma victims. The optimal decision instrument developed in this project could still safely decrease tomographic imaging of blunt head injury victims by more than 12%.

Quality assurance reviews (comparing patients enrolled in the study against patients undergoing emergency computed head tomography) indicate that the clinicians are able to evaluate and enroll patients with 98% compliance. Discussions with site physicians and coordinators indicate that our data collection is simple and efficient, makes minimal demands on physicians, and is well tolerated in the clinical environment. Although we have the ability to terminate data collection at any site that is not compliant with protocols, we have had to take this action at only two institutions. Thus, most study sites have little difficulty implementing our protocols, and they are able to complete the evaluations in a timely and reliable fashion.

Our computerized data transfer and concatenation system is reliable and efficient. Computer problems occur infrequently and are usually corrected by remote reconfiguration of the problem computer. Rare hardware problems have been corrected by over-night transfer of redundant computers to serve affected sites until permanent hardware could be repaired. Data loss due to hardware and software problems has been limited to approximately a dozen cases.

**3.2 - Sample size calculations.**

To be clinically reliable, the combined selective criteria must satisfy two requirements. First, intracranial injuries requiring neurosurgical intervention must never occur in blunt head trauma victims identified as risk free by the instrument. This is equivalent to requiring a 100% negative predictive value. Second, every blunt trauma victim with a significant intracranial injury must exhibit at least one of the risk criteria. This requirement implies that the sensitivity of decision instrument be 100%.

Verifying negative predictive value and sensitivity at an absolute level is not statistically possible with a finite sample size. However, it is possible to estimate limits for the true values of these proportions using exact statistical relationships. The lower confidence limit of a proportion (such as negative predictive value or sensitivity) is related to the total size of the study population. The quantitative relationship is expressed by the following equation [48]:


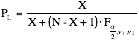


Where:

P_L_ = Lower confidence limit for the proportion being studied (i.e. the lower confidence limit for the negative predictive value or sensitivity of selective criteria).

X = Number of times the event of interest is observed.

N = Total number of observations.

α = Statistical confidence value (Set to 0.05).

ν_1_ = Degrees of freedom for the numerator; ν_1_ = 2 [ N - X + 1 ]

ν_2_ = Degrees of freedom for the denominator; ν_2_ = 2 X

F_α/2,ν1,ν2_ = One tailed value of the F-distribution with ν_1_ and ν_2_ degrees of freedom.

Validating a given P_L_ to a 95% level of statistical certainty implies that α = 5% (or α = 0.05). The main hypothesis of this study states that selective criteria will detect all intracranial injuries requiring neurosurgical intervention in blunt head trauma victims without missing any such injuries. Thus, for purposes of sample size calculations, for either negative predictive value or sensitivity, the event of interest (presence of one of the risk criteria) should occur with every observation. Hence, X = N.

The degrees of freedom for the numerator and denominator are then given by:

ν_1_ = Degrees of freedom for the numerator; ν_1_ = 2 [ N - X + 1 ] = 2 [ N - N + 1 ] = 2

ν_2_ = Degrees of freedom for the denominator; ν_2_ = 2 X = 2 N

The expression for the lower confidence limit then becomes:


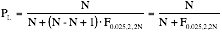


As discussed previously, practical concerns regarding radiographic charges and radiation health effects suggest that selective criteria will still remain cost effective and reduce mortality and morbidity even when missing 10 out of every 1,000 injuries. This implies that negative predictive value need only be verified to a 99.0% lower confidence level, or equivalently, P_L_ = 0.99. Substituting this value in the lower confidence limit equation yields the following algebraic equation for intracranial injury population size N;


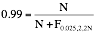


The numerical solution to this equation is N = 368. Thus, to confirm that the decision instrument has a negative predictive value of at least 99.0%, the study must enroll 368 head injury victims who have none of the risk criteria, and none of whom have intracranial injuries requiring neurosurgical intervention. Since approximately 10% of head trauma victims are free of the risk criteria, this derivation would require a population of 3,680 head trauma victims. Unfortunately, confirming that the instrument has a 99.0% sensitivity requires studying 368 intracranial *injury* victims. Surgically significant intracranial injuries occur in only 1.2% to 1.4% of head trauma cases. Assuming a serious injury prevalence of 1.2% implies that this study will need to prospectively evaluate 31,000 blunt head injury victims. Consequently, study size is driven by the requirements of sensitivity.

**4. EXPERIMENTAL DESIGN AND METHODS**

**4.1 - Experimental Design**

The study will be conducted as a multicenter prospective evaluation of blunt head trauma patients undergoing computed head tomography. Participating study centers encompass a range of acute care facilities including academic trauma centers, community trauma centers and community emergency departments. Surveying a wide range of hospitals will make the study results generalizeable the majority of mild head injury victims.

The study will use an experimental design similar to that implemented in the previous study *Selective Computed Head Tomography in Blunt Trauma* (PHS Grant No. R01 HS09699). This computed tomographic derivation study is particularly important to the current proposal because it has already evaluated the use of a multicenter network needed to conduct the blunt head injury validation research. In addition, both studies use very similar methodology and protocols. Thus, the core methodology for evaluating blunt head injuries has already been tested in the derivation study. Personnel trained for the earlier study have become experienced with this methodology and can be quickly updated to the new protocols needed for the validation study. Most importantly, the derivation study demonstrates that the methodology for the current proposal is feasible and can be conducted in the clinical environment. The derivation study involved over 800 physicians, as well as a similar number of supporting technicians and nurses, working at 21 institutions, and accumulated over 600 cases per month.

**4.2 Protocol Overview**

Emergency physicians working in affiliated study centers will collect survey data on all blunt head trauma victims undergoing computed head tomography. In stable patients, survey data will be collected prior to radiographic imaging. In unstable patients, clinicians will be expected to complete data collection without delaying patient care, whenever possible, and will formulate answers to data questions, even if not recorded, prior to radiographic review. Tomographic imaging of individual patients will be performed only after data collection is complete and a radiographic request has been issued. Radiographic requests will only be available after a complete data form has been submitted.

Information collected on each patient will include demographic information, select elements of history and physical examination and results of computed head tomography. Data from each study center will be captured and stored on a computer database for eventual concatenation with data from other centers.

All radiographic studies will be reviewed by clinical radiologists at each participating center. Injury determination and classification will be based on final radiological findings. Injury type and location will be recorded for all patients having significant head injuries. The hospital records of patients who have positive CT imaging will be reviewed to ascertain whether their injuries required neurosurgical intervention. Radiographic diagnoses and need for neurosurgical intervention will then be concatenated with survey data on a case-by-case basis to form the final study database.

**4.3 - Methods**

**4.3.1 - Structure of the Multicenter Study and General Administration**

The Principle Investigator and core study administration will be located in the UCLA Emergency Medicine Center. A total of 4 hospitals will participate as study sites. Each participating institution will be represented by an emergency physician who will serve on the project steering committee and act as a site liaison. These individuals will be responsible for implementation of study protocols at that institution, and be responsible for obtaining a waiver from their respective institutional review boards and notifying the principle investigator of any protocol problems or other difficulties encountered during the study. Each site will also be staffed by a study coordinator who will be responsible for collecting and concatenating survey responses, assembling and transmitting data to a central repository, and performing quality assurance checks at the site.

In addition to the participating centers, there will be core personnel at UCLA who will monitor data collection from the centers, perform central concatenation of survey data and radiographic data, and perform data analyses. This core team will consist of the Principal Investigator, one project design member (co-investigator), a radiologist, one study coordinator, and a small team of research assistants. This team will be under the direct supervision of the Principal Investigator. Appendix - A details the administrative structure of the study and lists participating hospitals and representatives for each affiliated institution. The hospitals are listed as either 1- University based private hospital, 2 - Community based private hospital, or 3 - Public hospital.

**4.3.2 - Data Collection and X-ray Ordering**

**4.3.2.1 - Patient Entry**

Every blunt trauma victim presenting to a study center will undergo a clinical evaluation. The examining physician will determine if computed head tomography is indicated, using whatever criteria they deem appropriate. Physicians will be informed that it is not currently possible to exclude intracranial injuries in patients who present with any of the risk criteria, and they will be encouraged (but not required) to obtain radiographic imaging on any blunt head trauma victims presenting with one or more of these criteria (see Appendix - B for a sample data entry screen and listing of criteria). Conversely, the clinicians will also be informed that the criteria have yet to be proven reliable, and that imaging should be considered for any patient suspected of harboring an intracranial injury. There will be no formal requirement to obtain or exclude tomographic imaging of any patient. Physicians will be free to order or omit computed head tomography at their discretion.

Data capture will occur each time a request for computed head tomography is made. Tomographic imaging will be performed only after a computer generated radiographic request is presented to the tomography technician. Tomographic requests will not be available until the examining physician completes a full assessment and records the survey responses. This strategy assures that a complete data set will be captured on each patient prior to radiographic imaging. Several of the participating sites use computer based automated entry systems to order radiographic imaging. Previous experience reveals that these systems can be easily modified to adhere to our study protocols, including the collection and de-identification of survey data and imaging results. Because these systems are automated, they do not print imaging requests, but can be programmed to issue requests only after survey data has been entered. Our two prior studies reveal that these systems are easily configured, very reliable, and easier to maintain than standard systems using printed requests.

Because computer generated requests will be required for all emergency department head tomographs, patients who present with non-traumatic indications may be enrolled in the study. Physicians will be required to indicate the non-traumatic nature of these evaluations. To assure compliance with the methods of this proposal, clinicians will still be required to enter relevant study information. This information will be retained in a separate database, and will be analyzed separately from data on blunt head injury victims. This information may be useful in developing selective indications for non-traumatic computed head tomography. However, this information is not relevant to the hypotheses being tested in this study.

Imaging requests may be generated prior to complete data entry in physiologically unstable patients. Clinicians will ultimately complete data entry in these patients, but complete data entry may occur after computed head tomographs have been reviewed. In such cases, the data entry for the case will document the physiologic instability and non-concurrent nature of the data collection. In our previous study, clinicians classified 8.7% of patients as unstable (including 17.5% of patients with intracranial injuries), but were able to complete assessments on all patients. In this regard, the study protocol allowed clinicians to provide expeditious care to even the most severely injured patients, while having a negligible impact on data collection.

Data will be collected on all blunt head injury victims entered into the study. Inclusion will not depend on age, sex, race or ethnic origin. Specific information regarding race will be collected to determine what effect, if any, race may have in evaluating patients for risk of injury. Demographic analyses will be performed on age and sex data. Study sites (Appendix A) have been selected to represent a broad spectrum of medical environments. These sites are expected to enroll a diverse population containing all races and both sexes. All sub-populations will be represented in the study in the same proportions as they present with blunt head injuries.

**4.3.2.2 - Assessing the potential for work-up bias**

It is possible that a patient with a significant head injury could exhibit none of our study criteria *and* not be selected for tomographic scanning. Such a case would represent an undetected false-negative event; all combinations of our criteria would fail, and we would be unaware of the failure. Such cases would create work-up (verification) bias and lead to erroneous estimates of sensitivity and negative predictive value. It is not possible, within reasonable budget limitations, to track all blunt head injury victims for such an occurrence, however, it is possible to tract a screening subset to determine whether false negative events occur and to estimate the potential magnitude of work-up bias. Thus, the presence or absence of each screening criterion and long-term outcome will be determined on a cohort of 368 consecutive blunt head injury patients who present to the Ronald Reagan UCLA Medical Center, but do not undergo CT head imaging.

The clinician caring for these patients will invite them to participate in a long-term outcome study. Research assistants, present in the emergency department on a continuous basis, will contact interested patients (or their caretakers) during their initial visit and obtain informed consent to participate in a structured telephone survey to take place three months after the date of injury. Patients who agree to participate in the study will be asked to provide a preferred time and phone number for the follow-up survey. Patients who do not have phones will be given the option of returning to the emergency department for the survey. Additional details of the work-up bias assessment are included in later sections of this proposal.

**4.3.3 - Data Collected**

Data collected by the study include reason for tomography (blunt trauma, other), patient stability (stable, unstable), date of injury, patient’s date of birth, race and sex. Clinicians will assess each patient for the presence or absence of the following criteria: 1 - signs of basilar, depressed or diastatic skull fracture, 2 - scalp hematoma, 3 - neurological deficit, 4 - abnormal level of alertness, 5 - abnormal behavior, 6 - persistent or forceful vomiting, 7 - coagulopathy. Age will be determined by referencing the patient’s date of birth to determine if they are of age greater than 65 years. Clinicians will also assess the patient’s Glasgow Coma Scale score, whether the patients experienced a dangerous mechanism of injury, or whether the patient exhibited more than 30 minutes of amnesia before impact. Appendix - B contains a sample data collection sheet, and Appendix - C gives precise definitions and instructions for classifying physical findings. Each computer record will also carry embedded information indicating the institution producing the data.

We will prepare special education kits (similar to the ones we used in the previous head injury study) with scripts, slides and overheads to assist site physicians in training their clinicians in our study protocols. The clinicians will be introduced to the candidate criteria by reading the descriptions provided in Appendix - C. We will intentionally avoid exposing the clinicians to any more detailed training regarding the definitions and assessment of the candidate criteria. The rationale for this action relates to the ultimate implementation of our clinical decision instrument. Past experience shows that it is possible to develop and validate clinical decision instruments that have exceptional performance when implemented by specially trained and highly skilled evaluators. These same instruments subsequently exhibit poor performance when implemented by less skilled practitioners in the “real world” [59]. It is our goal to assess the performance of practicing clinicians who have not received special training in criteria assessment, and may have gained an understanding of the decision instrument by reading the medical literature. Thus, we would like to validate our decision rule using physicians who’s exposure to the criteria is similar to that of a physician who might have encountered the instrument while reading the medical literature. This is an approach similar to that used in validating our cervical spine decision instrument, and in the derivation phase of this study. Subsequent evaluations have shown that clinicians can reliably and consistently assess the presence or absence of the criteria [60,61,71].

It is possible that clinicians may not be able to evaluate individual criteria in some patients. For example, it may not be possible to perform a detailed neurological exam and assess motor strength in an individual who has multiple extremity fractures and is unable to cooperate. Clinicians can indicate such occurrences by designating the presence or absence of the individual criterion as “unknown”. The overall decision instrument will treat these “unknown” responses as abnormal responses. Individual risk criteria will be classified as absent only if they can be tested and found to be absent. Consequently, the decision regarding the ultimate need for tomography is based on measuring individual criteria and determining that they are *all absent* in a given individual. If a patient exhibits any of the criteria, or if one or more of the criteria cannot be assessed, the individual will be labeled positive by the decision instrument. Such patients may harbor a significant intracranial injury and should be considered candidates for tomographic scanning. Clinicians are not to use the “unknown” designation in lieu of an adequate evaluation. Our experience with the cervical spine and head injury derivation studies reveals that study physicians use this designation appropriately.

**4.3.3.1 - Work-up bias assessment**

Clinicians will assess the presence or absence of each study criterion for all blunt head injury victims presenting to the Ronald Reagan UCLA Medical Center, including a cohort of 368 patients who do not undergo tomographic imaging during their initial visit, but agree to participate in the long-term outcome evaluation. Research assistants will have the evaluating clinicians record their criteria assessments (onto paper forms – see Appendix B) as soon as they have obtained each patient’s informed consent. The research assistants will also record a coded study number along with each patient’s age, sex, race, and date of injury, as well as a preferred time and phone number for the follow-up survey. Patients who do not have phones will be given the option of returning to the emergency department for their survey, and the patient’s address will be recorded on the survey form in lieu of a phone number. A reminder to participate in the survey will be mailed one week prior to the survey date to each patient who elects to return to the emergency department for the follow-up survey.

The research assistants will maintain a calendar containing the call-back schedule for the follow-up survey. At the time of enrollment, the patient’s coded study number will be entered into the calendar on the date and time corresponding to the scheduled appointment for the long-term survey (i.e. three months after the patient’s visit). The exact scheduling of these appointments will be negotiated between the research assistant and the patient, with the proviso that the survey cannot take place prior to 90 days after the injury. The research assistants will be further encouraged to complete each survey within 100 days of the patient’s injury.

The research assistants will contact each patient at the time previously scheduled for their follow-up survey. If the patient is unavailable at the scheduled time, the assistants will attempt to re-contact the patient using the following schedule: two times on the day of the interview, once daily for five days after the date of the interview, and once weekly for three weeks after the date of the interview. Upon successful contact the assistants will either complete the survey or reschedule the interview depending on the patient’s preference. Contact will be abandoned, and the patient deleted from the work-up bias evaluation if the assistants are not able to contact the patient within four weeks of the scheduled survey date.

Once contact is established, the research assistants will administer the survey using the script presented in Appendix - D. Patients, or the care-takers of incapacitated patients, will be asked whether they sought care at another facility after their discharge, whether they received any x-rays or imaging studies, and if so, what type, and whether they received any treatments, hospital admission or brain surgery as a result of the injury. Patients will be considered to have had a significant intracranial injury if they had a neurosurgical procedure performed as a result of their injury. After the completion of each survey, the survey responses will be paired with matching criteria assessments to form a complete data record for each patient.

Criteria assessments, survey results and the contact information obtained on each follow-up patient will be retained in a secure, locked file that will be indexed using the coded study numbers. Data from this file will only be accessible to the research assistants and study administrators. Information from completed surveys and matching criteria assessments will be entered into a computer database for long-term storage, transmission and analysis. Data will be entered into the computer using a redundant input system where each data element is entered twice. This system will protect against keyboard and entry errors, and help ensure data integrity. The computer data will be indexed using the coded study numbers and will not contain information that could be used to identify individual patients. The final record for each patient will contain the patient’s age (if less than 90 years, or a 90+ designation for patients older than 90 years), sex, race, date of injury, presence or absence of each criterion, date of follow-up, whether the patient sought additional care, whether the patient received imaging (and if so, the type of imaging: skull films, CT, MRI), whether the patient received any treatment, whether the patient was admitted to the hospital for their brain injury, and whether the patient underwent a neurosurgical intervention (and if so, what type). All contact information and any linking patient identifiers will be destroyed once follow-up information has been obtained.

Research assistants will make weekly backup copies of the computerized database, at which time the data will be reviewed for exceptional events. Cases will be considered to represent false-negative events if the patient was classified as low risk by the decision instrument, yet required neurosurgery. Cases will be counted as true-positives if they required neurosurgery and were not classified as low risk by the decision instrument. The error in sensitivity produced by work-up bias will be determined by computing the difference between the true sensitivity, as measured among the entire cohort of head injured patients presenting to the UCLA site during the work-up bias assessment, and the sensitivity measured among patients from this site who were selected for imaging. The exact formula for this calculation is as follows:


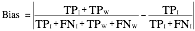


Where:

TP_I_ = Number of imaged individuals requiring neurosurgery and labeled positive by the decision instrument (number of true-positives among imaged patients).

FN_I_ = Number of imaged individuals requiring neurosurgery and labeled negative by the decision instrument (number of false-negatives among imaged patients).

TP_W_ = Number of un-imaged individuals requiring neurosurgery and labeled positive by the decision instrument (number of true-positives among un-imaged patients).

FN_W_ = Number of un-imaged individuals requiring neurosurgery and labeled negative by the decision instrument (number of false-negatives among un-imaged).

Confidence limits for this measure will be calculated by applying boot-strap estimation to the entire UCLA cohort (both imaged and un-imaged patients) [73]. Estimates of the work-up bias associated with negative predictive value and specificity will similarly be calculated by computing the absolute difference of these values among imaged patients and the entire cohort.

These methods are similar to those used in assessing the potential for work-up bias in our previous derivation study (PHS Grant No. R01 HS08239). The methodology has shown to be reliable, and we were able to obtain follow-up survey information on 98% of the patients who agreed to participate in the study.

To verify the potential for work-up bias to with 1.0% requires a population of 368 head injury patients who do not receive CT imaging during their initial presentation. Thus we will enroll 368 consecutive patients in the work-up bias portion of the study.

**4.3.4 - Patient Consent**

For most patients, this observational study collects information routinely obtained in the evaluation of blunt head trauma victims. This information should be documented in each patient’s medical record, although past experience has shown that physicians are not always meticulous in detailing their findings (this is part of the motivation for performing this study as opposed to a chart review). The study does not alter medical care, and therefore poses no risk to the welfare of patients or place them at any additional medical risk. In addition, the data will be indexed by study numbers assigned to each case at the time of enrollment, data will be recorded in such a manner that subjects will not be able to be identified, either directly or through identifiers linked to the subjects. These conditions comply with the terms for waiver of informed consent. The protocols in this project are compliant with new HIPAA regulations and have undergone IRB review and an appropriate waiver has been granted. Each participating institution will be expected to obtain an individual waiver of informed consent (this requirement is identical to the procedures in place for our previous study of *Selective Computed Head Tomography in Blunt Trauma* (PHS Grant No. R01 HS09699).

Study index numbers will be used to link radiographic results and the need for neurosurgical intervention to survey data. This can be accomplished in a manner identical to that used in our earlier studies using the following procedure. At the time a radiographic request is printed, an additional sheet is printed. The upper left corner of the sheet contains the patient’s medical record number, while the lower corner of the sheet contains the study index number. These sheets will be collected by site coordinators. By examining the upper left hand corner of each page, the coordinators will know which radiographic studies to retrieve. Once the radiographic study has been collected for a patient, the regional coordinators will place the lower right hand corner of the study sheet (containing the study index number) over the patient identifying information on the tomography report. The tomography report will then be photocopied with the study index number covering all identifying patient data, thus effectively redacting all identifying information from the tomography report. If the tomography report reveals an intracranial injury, the patient’s medical records for the associated visit will be reviewed, and the redacted tomography report will be modified by including a notation regarding the need, if any, for neurosurgical intervention. The redacted photocopies will then be forwarded to UCLA. Once the tomographic reports and need for neurosurgical intervention have been collected, redacted and photocopied, the sheets containing both patient medical record and study number will be cut in half to yield a one leaf with patient medical record number, and a separate leaf with the study index number. A mark will be placed on the leaf containing the patient medical record number of any patient requiring neurosurgical intervention. The study index numbers will be destroyed, while the medical record numbers will be retained for quality assurance reviews (see below). All sheets will be destroyed on study completion.

Informed consent will be obtained only on patients participating in the long-term follow-up survey at the Ronald Reagan UCLA Medical Center. Clinicians will assess the presence or absence of each study criterion for all blunt head injury victims presenting to this center. Cases selected for CT imaging will be entered into the study under the waiver of informed consent using the process described above. Patients who do not undergo tomographic imaging will be invited by their physicians to participate in the long-term outcome evaluation. Research assistants will provide information about the study and assist in obtaining informed consent from patients who express interest in participating in the study. Patients will not be enrolled in the follow-up study unless they (or their surrogates) provide informed consent.

**4.3.5 - Tomographic Orders**

Once clinical data has been entered into the computer, tomographic request will be generated. These requests will be printed on the radiographic forms or computer systems currently in use at each institution.

**4.3.5.1 - Radiographic Imaging**

**4.3.5.1.1 - Radiographic Evaluation**

All patients entered into the main study will initially undergo computed head tomography. Cases will be excluded from the study if the tomography is incomplete and all scan slices are negative. Initial tomography may be supplemented by plain radiographs, additional tomography, angiography or any other imaging study deemed necessary by either ED physicians, neurosurgeons, or radiologists.

**4.3.5.1.2 - Injury Diagnosis**

All radiographs will be reviewed by clinical radiologists at the individual study centers, who will be unaware of the patient’s classification by the decision rule. The diagnosis of significant intracranial injury will be based on final radiological interpretation of emergency department studies as well as any additional studies performed in the in-patient setting. Cases for which the initial diagnosis of “intracranial injury” or “no intracranial injury” proves incorrect will be reclassified to the appropriate group.

Copies of final computed head tomography readings will be collected on every patient in the study. The collected reports will include the final readings of every computed head tomography procedure performed on every patient. These reports will be collected on a weekly basis. Each report will be abstracted to determine whether a significant intracranial injury was detected by the procedure. Additional radiological studies of the head (magnetic resonance imaging, angiography, etc.) will also be collected and abstracted to ascertain which modalities produced diagnostically specific information. Final diagnoses of significant intracranial injury will be made on the basis of these reports and the need for neurosurgical intervention. In the event of ambiguous reports, Doctor Michael Zucker will review all of the available radiological studies and make a final determination of injury status. Data abstracted from each report will be concatenated with the computer survey data to form the final study database.

For operational purposes, a patient will be considered to have a positive computed tomogram if they exhibit any of the following findings [72]:

Mass effect or sulcal effacement

Signs of herniation

Basal cistern compression or midline shift

Extensive subarachnoid hemorrhage

Significant epidural or subdural hematomas (greater the 1.0 cm in width, or causing mass effect)

Significant cerebral contusion (more than 1.0 cm in diameter, or more than one site)

Hemorrhage in the posterior fossa

Intraventricular hemorrhage

Bilateral hemorrhage of any type

Depressed or diastatic skull fracture

Pneumocephalus

Diffuse cerebral edema

Diffuse axonal injury

Injuries such as isolated linear or basilar skull fractures, solitary small cerebral contusions, localized subarachnoid hemorrhage less than 1 mm thick, thin subdural hematomas less than 4mm thick, isolated pneumocephaly, and closed depressed skull fractures that do not violate the inner table will not be considered to be significant injuries [63]. Coincidental or congenital abnormalities will also not be considered to be significant intracranial injuries. Chronic subdural hematomas, without evidence of acute change, will be considered to be coincidental injuries and will not be considered to be significant acute injuries. Chronic subdural hematomas that exhibit any recent change, or those that are of indeterminate age, will be considered to be acute significant injuries.

Research assistants, blind to classification by the decision rule, will review the medical records of patients with positive tomographic scans to determine whether or not the patient received neurosurgical intervention. The need for neurosurgical intervention is defined as any of the following within seven days of the initial injury [63]:

1 – Death due to intracranial injury.

2 – Intubation for management or support of head injury.

3 – Craniotomy, elevation of skull fracture or intracranial pressure monitoring.

Cases involving death or intubation will be reviewed by the physician liaison to determine, on the basis of their best clinical judgment, whether there was a causal relationship between the event and the patients intracranial injury. The liaisons will be instructed to consider these events as causally related to intracranial injury unless there is compelling evidence to the contrary. Patients will not be considered to have received neurosurgical intervention if their death is clearly due to causes unrelated to their head injury, or if they were intubated solely to expedite care.

**4.3.5.2 - Radiographic Request Generation**

Radiographic request forms will be handled by dedicated computer systems. These devices will submit requests for computed head tomographic imaging each time a complete data record is entered. This will aid in protecting the integrity of the data and prevent accidental data entry or tampering.

**4.3.6 - Database Management**

A database application will be used to display the data questionnaire, provide input fields for survey responses, and direct storage and management of study data. This system will employ a redundant entry system that requires each response to be recorded twice. Discrepancies between responses will generate a prompt that will require the data to be re-entered until concordant responses are recorded. This process will help minimize the potential for data corruption from keyboard entry errors. Our database applications have been previously developed, tested and implemented in the prior studies on selective criteria in cervical spine injury and selective tomographic imaging in blunt head trauma. Captured data will be initially stored in a database file on the microcomputer hard disk drive until the next weekly data transfers and concatenation. In addition to data transfers, data will remain in each system, to serve as an ultimate backup, until completion of the entire study. Once a complete survey form has been entered into the database it will be unalterable by the affiliated site hospital. Error correction will follow the protocols described below. The examining clinicians will have access only to survey entry forms, while site liaisons and coordinators, and UCLA data analysts will be able to examine and transmit entire data sets.

A multi-functional database management application and programming language will be used as the main database management tool to create, store, maintain and analyze data records. This application will be used to program file maintenance utilities (i.e. concatenating programs, search routines, error detection programs, etc.), and its analytic capabilities will be used extensively in data analyses.

**4.3.7 - Data Analysis**

The primary outcome for the study is the point measure and associated 95% confidence interval for the sensitivity of the decision instrument in identifying patients who require neurosurgical intervention. Additional operator characteristics of interest for this population include the negative predictive value and specificity. Secondary outcomes include the sensitivity, negative predictive value and specificity of the instrument in detecting patients who harbor clinically significant intracranial injuries as described by the criteria defining positive CT imaging (see 4.3.5.1.2 - Injury Diagnosis above). A further secondary goal of the study is to determine and compare the operator characteristics of both the NEXUS head CT instrument, as well as both forms (“high risk” and “medium risk”) of the Canadian head CT instrument, among patients who meet the inclusion and exclusion characteristics for classification by the Canadian rules.

**4.3.7.1 - Incomplete Records**

Data from individual study centers will be concatenated into a single database at UCLA. Study records will be reviewed on a twice-monthly basis and regional coordinators informed of missing radiographic reports. The coordinators will be prompted to search for missing cases (as referenced by the study index number). Records of all patients will be reviewed at the close of the study, those lacking tomographic reports will be deleted.

**4.3.7.1.1 - Stable patients**

Because complete survey data will be required on all stable patients prior to computed tomographic requests, these records will only be deleted if radiographic reports and need for neurosurgery are missing.

**4.3.7.1.2 - Unstable patients**

Unstable patients make up a small minority of minor blunt head injury victims. The exact proportion varies from hospital to hospital. This makes it impossible to accurately estimate how many of these patients may be entered into the study. Because physicians may defer complete data entry on unstable patients, it is possible that final records for some of these patients might be incomplete. However, in our selective cervical spine and previous head injury studies we found that less than 10% of the study patients were classified as unstable, and all of these patients had complete criteria assessments. Thus, missing data from unstable patients is unlikely to be a significant problem in this validation study. In addition, physicians will be required to supply a minimal amount of demographic information on these patients prior to tomographic request generation, and are requested (and required by protocol) to complete data entry on all patients (even though this data entry may be non-concurrent). Records containing incomplete data will not be used in validating the decision instrument. These entries *will* be used in descriptive analyses on the demographics and injuries patterns associated with mild head trauma. Cases missing tomographic reports and information regarding the need for neurosurgical intervention will be deleted from the study.

**4.3.7.2 - Mathematical calculations**

The following mathematical forms will be used in statistical analyses:

**4.3.7.2.1 - Sensitivity, specificity and negative predictive value**

Sensitivity, negative predictive value and specificity will be used to determining the performance of the decision instrument. These measures will be calculated using the following standard formulae:

Sensitivity
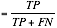
 Negative Predictive Value


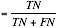


Specificity


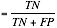


Where:

TP = Number of individuals with intracranial injury labeled positive by the decision instrument.

FN = Number of individuals with intracranial injury labeled negative by the decision instrument.

FP = Number of individuals with no intracranial injury labeled positive by the decision instrument.

TN = Number of individuals with no intracranial injury labeled negative by the decision instrument.

Confidence intervals for sensitivity, specificity and negative predictive value will be determined using exact limits for proportions [48]:


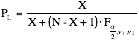


Where:

P_L_ = Lower confidence limit for the proportion being studied (i.e. the lower confidence limit for the negative predictive value or sensitivity of selective criteria).

X = Number of times the event of interest is observed.

N = Total number of observations.

α = Statistical confidence value (Set to 0.05).

ν_1_ = Degrees of freedom for the numerator; ν_1_ = 2 [ N - X + 1 ]

ν_2_ = Degrees of freedom for the denominator; ν_2_ = 2 X

F_α/2,ν1,ν2_ = One tailed value of the F-distribution with ν_1_ and ν_2_ degrees of freedom


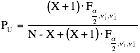


Where:

P_U_ = Upper confidence limit for the proportion being studied.

X = Number of times the event of interest is observed.

N = Total number of observations.

α = Statistical confidence value (Set to 0.05).

ν′_1_ = Degrees of freedom for the numerator; ν′_1_ = 2 [ X + 1 ]

ν′_2_ = Degrees of freedom for the denominator; ν′_2_ = 2 [ N - X ]

F_α/2,ν′1,ν′2_ = One-tailed value of the F-distribution with ν′_1_ and ν′_2_ degrees of freedom.

The sensitivity and negativity predictive value will determine whether the decision instrument reliably identifies all patients with significant intracranial injuries. The true-negatives will reveal the number of patients who can safely forego radiographic imaging. Comparisons between injured and non-injured groups will be made by chi-square testing, or Fisher's exact test if any cell has less than 5 subjects. Bonferroni or other statistical adjustment will not be made for analyses performed for descriptive purposes.

**4.3.8 - Quality Assurance**

**4.3.8.1 - Protection of Survey Data**

The site liaisons will be charged with verifying compliance with study protocols at their institution. Specifically this will require the liaisons to verify that survey data on all patients who undergo computed head tomography is accurately entered into the computer database.

Coordinators will review the tomography logs on a weekly basis to verify that all blunt head trauma victims who have undergone computed head tomography have also been entered into the computer database at their site. Any omitted cases will be discussed with the emergency physicians for that site and an attempt to identify any protocol errors will be initiated. Omissions due to personnel non-compliance will result in counseling of the involved parties. Omissions due to mechanical, protocol or system problems will be handled by consultation with the principal investigator at UCLA. The UCLA investigator will then determine whether protocol corrections are needed and will direct the implementation of any changes. To minimize corruption of the study data, any cases omitted from the database due to personnel or system failures will be excluded from the study.

Site liaisons will also be responsible for verifying that data is being accurately entered into the computer. They will observe relevant cases as they are processed by the emergency department, and will verify that each patient is seen by a physician, that survey data is entered into the computer, and that computed tomography is obtain as specified by the protocol.

Quality assurance checks will also be randomly performed by the liaisons to verify that physicians are adhering to examination guidelines. The physician liaison will be responsible for verifying that appropriate physical examinations are being performed and recorded on all patients presenting to their site. Any violations of protocol or incomplete examinations will result in verbal counseling of the involved parties and re-education in proper technique. Repeat flagrant violations will result in suspension of data collection by the involved physician. All records produced by a suspended physician will be reviewed and any suspect cases will be deleted. These physicians will still be able to obtain emergent tomographic imaging by attaching their name and the patient’s hospital number on a pre-generated waivers. These waivers will be enable CT requisition without survey entry, and will enable technicians to perform the needed imaging. Liaisons will collect the waivers during their routine quality assurance assessments and ensure that they are being used sparingly, and only by the suspended physician. Misuse of the waivers will result in termination of data collection at the involved institution.

**4.3.8.2 - Protection of Radiographic Data Integrity**

Preliminary diagnoses of "no injury" will be confirmed in two ways. First, quality assurance logs, which contain lists of significant radiological findings that were missed on preliminary reading, will be reviewed by the site liaisons during routine evaluations. Any revisions found on these surveys will be transmitted to UCLA where they will be incorporated into the study database. Second, the diagnoses of all blunt trauma patients admitted through the emergency department and discharged from the hospital during and up to three months after each study period will be searched and all intracranial injury cases identified. The charts of such patients will be reviewed to determine if the case was included in the study (using the patient number previously printed on the redacting forms), and if so, whether the case was classified as having an intracranial injury (based on whether the leaf was marked). The liaisons will notify core UCLA personnel of any cases of missed injury detected in this fashion. These cases will be reviewed by the site liaisons to determine, based on their best judgment, whether they involve protocol errors, and whether they represent a false-negative event (patient with a head injury, but “low-risk” according to the decision instrument). Site coordinators will notify core UCLA personnel of their findings. Reviews of the quality assurance logs and discharge diagnoses will be performed by the site liaisons on a bi-monthly basis.

**4.3.8.3 - Error Processing and Protection of Data Integrity**

It is possible that a clinician may make an error in a data collection field that is not realized until after the survey has been added to the database. In the event of such an incident, the clinician will be required to submit a corrected survey. The original erroneous survey will remain on the computer database and will not be removed until the data is concatenated into the final data collection. All corrected entries will contain coding information designating the erroneous data they supplant. This strategy will ensure integrity of the database and prevent unauthorized tampering of results. In addition, after final concatenation of survey data and tomography results, the record of any patients who did not have tomographs will be deleted from the study.

As indicated above, data collection may be by-passed on unstable victims. Such cases will be excluded from the study if the data questionnaire is not complete. Clinicians will be informed that an initially incomplete form may be completed at a later time using the same technique as described for error correction. Again, the correct and complete form will contain coded linkages to the incomplete or erroneous data record, and as stated previously all data on physiologically unstable patients will be kept and processed through a separate database. Previous experience has shown that physicians rarely enter incomplete data sets on unstable patients. In our selective cervical spine and head injury studies, we found that physicians were able to complete criteria assessments and record their findings on all unstable patients.

Additional security and data protection will be accomplished through a keyed entry system with entry codes available only to the regional study coordinators and administrators. Individuals who do not have access keys will be able to enter study data using the screen entry program, but will not have access to any additional functions on the computer.

**4.3.8.4 - Equipment Failures**

Data collection at individual hospitals will be suspended in the event of computer system failure. If such a failure should occur, any retrievable data will be transmitted to UCLA for concatenation at the earliest possible opportunity. Site liaisons will be responsible for identifying equipment failures and initiating appropriate corrective action. During equipment failures, the affected institution will be allowed to perform data collection using pre-printed paper survey forms and will need to obtain radiographic imaging using their pre-study protocols. Information collected on printed forms will be entered into the study database at the earliest possible opportunity.

**4.3.8.5 - Study Site Termination**

If an individual study site should withdraw from the study, or otherwise be unable to participate in data collection, the site will be removed from the study and all collected data retrieved. A new site will then be selected from a list of alternative hospitals. The site will be chosen on the basis of geographical proximity, hospital type (community vs. university vs. county) and patient population.

**4.3.9 - Difficulties and Limitations**

**4.3.9.1 - Sensitivity and Negative Predictive Value**

Errors in the estimation of sensitivity and negative predictive value of the screening strategy could bias the internal validity of this study. Bias in these estimates could occur through misclassification of patient injury status, or if doctors change their answers after tomographic review and diagnosis. The diagnosis of injury will be based on the final tomographic interpretation and need for neurosurgical intervention, and in the majority of patients, absence of injury will be determined by a single computed tomographic series. Thus, true sensitivity will depend on both the sensitivity of selective criteria as well as the negative predictive value of standard tomographic imaging. However, it is not the purpose of this study to determine the optimal method of radiographic evaluation. If an injury is missed on standard computed tomography, the method of patient selection is irrelevant since such cases would be misidentified even if tomography were performed on all patients. Thus, the measured sensitivity is the parameter of interest. Bias due to alteration of data forms will be eliminated by requiring data to be entered into the computer prior to generation of tomography requests. This leaves the potential for misrepresentation of data in critically ill patients, but these patients will in general be excluded from the mild head injury status by virtue of their critical illness. Thus bias in sensitivity due to data alterations should be insignificant.

Errors in sensitivity and negative predictive value may also occur through biased enrollment. Practical concerns, such as cognitive impairment among head injury victims, make it impossible to obtain informed consent on all patients. To eliminate the potential for selection bias we instituted methodologies that allow us to conduct an observational study under a waiver of informed consent. This approach ensures that we will enroll all patients who undergo tomographic scanning (including those most likely to have intracranial injuries), but limits our ability to obtain CT scans on all blunt head injury victims. In this process, our methodology introduces the potential for workup bias. However, errors would occur only if a patient with a significant head injury exhibited none of our study criteria *and* was not selected for tomographic scanning. Such a case would represent an undetected false-negative event; the decision instrument would fail, and we would be unaware of the failure. Although such an event is theoretically possible, available studies indicate that essentially all significant head injury victims exhibit some symptomatology. As noted in our background discussion, there are no case reports describing significant intracranial injuries in an individual who does not exhibit at least one of our initial screening criteria. Furthermore, in our derivation study, we obtained follow-up evaluations on 1,266 patients who did not undergo computed tomography during their initial presentations. These evaluations revealed that a small minority of these patients ultimately received cranial imaging. However, none of these cases required hospitalization or neurosurgical intervention, and there were no cases of missed intracranial injuries or death among this cohort [65]. Thus, the potential for work-up bias is a theoretical possibility, but is of less practical concern. Furthermore, we will screen patient revisits and inpatient neurosurgical trauma admissions to identify patients who might produce such bias. Finally, as mentioned previously, we will obtain criteria evaluations and contact information for 368 consecutive blunt head trauma patients who present to the Ronald Reagan UCLA Medical Center and do not undergo emergent tomographic head imaging during their initial evaluation. We will contact these patients, or their family, three months after their enrollment to determine whether they were eventually diagnosed as having a significant intracranial injury or required hospitalization or neurosurgical intervention. The survey form used in these follow-up assessments is similar to the one used in the previous head injury study, and a copy of the survey form is presented in Appendix - D. Based on our previous experience, we estimate that we will be able to complete survey interviews on approximately 98% of the enrolled patients [65]. This process will effectively screen a large subset of patients who are not otherwise enrolled in the study, and will allow us to not only detect workup bias, but also estimate its magnitude. Thus, although we cannot completely eradicate the potential for measurement bias, our methodology makes it unlikely that it will be present without our knowledge.

**4.3.9.2 - Specificity**

Bias in the specificity measurements could result from misclassification of subjects, or from selection bias due to non-random exclusion of patients from the study. Such exclusions could produce a study population that does not represent trauma patients in general. If many patients omitted from the study have "no-risk" criteria then our measure of specificity will be too low; the converse is also possible. It is impossible to determine the direction of possible bias in our specificity measurements. Our specificity estimates, even if biased, would be very unlikely to change enough to substantially alter our conclusions. For example, if clinicians ordered tomography only for seriously injured patients, most of whom have at least one of the risk criteria, then the number of uninjured patients labeled negative by the criteria will be small and the specificity will be low. On the other hand, if clinicians order large numbers of tomograms on patients with no complaints, then the number of uninjured patients labeled negative by the criteria will be large and the specificity will be high. The first scenario would result in erroneously low estimates of cost savings using criteria, while the second scenario would over estimate savings, however, neither scenario would alter the number of patients who have injury and are identified as such by the criteria. Thus errors in specificity may affect cost-saving estimates but will have little influence on the validity of selective criteria. Furthermore, data collection at the Pittsburgh site will include imaged as well as un-imaged cases, and will provide a means of estimating the specificity of the instrument when applied to all blunt head injury patients.

**4.3.9.3 - External Validity**

The external validity of this study could be compromised through ambiguities arising from study population selection. Since individual physicians can decide whether some patients need tomography, our population could differ from that selected by a different group of physicians. Examining physicians may choose to "clinically clear" patients without documenting findings or obtaining radiographic imaging. Such patients are unlikely to harbor significant intracranial injuries, and elimination of this potential bias would, if anything, strengthen our results, by narrowing the confidence interval of the specificity of our low-yield criteria. Although the predictive values of the screening strategy may change when applied to different patient populations, the broad range of hospitals and clinicians used in the study should make the results generalizeable to a large number of blunt head trauma victims.

**4.3.10 - Work Schedule**

**4.3.10.1 - Start-up Period**

The initial weeks of the study will be used to procure required equipment and train personnel. This time will also be used to develop the data capture screens, modify the screen display tables, and set up and test the modified data collection system. Any data collected under study protocols during this initial period will be included in the final database.

**4.3.10.2 - Running Schedule**

Data collection will occur around the clock for the entire duration of the study period. Similarly, radiographic data will be collected on a continuous 24-hour basis. Data will be concatenated into the main study database as soon as it has been transmitted to UCLA. Radiographic reports will be collected, abstracted and concatenated into the final database on a weekly basis. Finally, each week the entire database will be analyzed for exceptional information that could result in the termination of the study (see “Duration of the study “ below).

Problems in data collection, hardware and software problems and other unexpected difficulties will be monitored and corrected on an ongoing basis. In addition, there will be a once a month review of over-all system performance and evaluation of ongoing problems. During this time protocol updates will be communicated to all centers, inefficiencies corrected and non-emergent problems addressed.

**4.3.10.3 - Duration of the study**

The study duration will be determined by the following:

The study will terminate if it encounters 10 cases having significant head injury that requires neurosurgical intervention, but labeled “low risk” by the decision instrument. Data collection will otherwise continue until the study enrolls 368 cases requiring neurosurgical intervention (we anticipate this may require as many as 31,000 total blunt head injury cases).

In the unlikely event that the study terminates without validating the clinical decision instrument, the data collected in the study will still be used to provide important information about blunt intracranial injury. This will include information on the demographics of head injury including overall prevalence, geographic and seasonal variations, and distributions by age, race and sex. Tomographic reports will yield important information on the different types of intracranial injuries, including prevalence and distributions among the various demographic groups. This information will be of use in emergency neurological and clinical assessment, and will prove useful in formulating future recommendations. Data on the presence or absence of the criteria in individual patients will be useful in assigning clinical significance to the criteria. This data will be analyzed by regression analysis to identify relationships between intracranial injury and clinical presentation. Such studies can aid in risk assessment and stratification of risk among blunt head injury patients. This information will in turn guide future studies that seek to improve the management of blunt head trauma victims. Finally, the failure to validate the decision instrument is an important finding on its own. It would suggest that clinicians must be extremely careful in evaluating head injury patients and that tomographic scanning may be indicated in many if not most of these individuals.

It is also important to recognize that this study will collect data on all patients undergoing emergent tomographic head imaging, including individuals who are imaged for atraumatic indications (i.e., seizures, severe headache, prior to lumbar puncture, etc/). Information collected from the evaluation of these patients will be combined with similar data collected in our derivation study. This information will not only be used to derive and validate imaging recommendations for these other presentations, but will provide valuable information on patient epidemiology and disease patterns in these groups. Much of this work will follow protocols similar to those outlined for blunt head injury patients, but a detailed description is beyond the scope of this proposal.

**4.3.11 - Specific Duties of Study Personnel**

The specific duties of study personnel are listed in Appendix - E.

**5. HUMAN SUBJECTS**

This methodology has been reviewed and waiver of informed consent granted by the UCLA Office for Protection of Research Subjects (UCLA - IRB) in association with the previous derivation phase of the study. Approval for the validation phase will be obtained prior to initiation of the study. Each individual study site will be required to obtain a consent waiver prior to enrolling any patients.

**5.1 - Risks to the subjects**

This observational study collects information routinely obtained in the evaluation of blunt head trauma victims. This information should be documented in each patient’s medical record, although past experience has shown that physicians are not always meticulous in detailing their findings (this is part of the motivation for performing this study as opposed to a chart review). The study does not alter medical care, and therefore poses no risk to the welfare of patients or place them at any additional medical risk. In addition, the data will be indexed by study numbers assigned to each case at the time of enrollment, data will be recorded in such a manner that subjects will not be able to be identified, either directly or through identifiers linked to the subjects. These conditions comply with the terms for waiver of informed consent. The protocols in this project have undergone IRB review and an appropriate waiver has been granted. Each participating institution will be expected to obtain an individual waiver of informed consent (this requirement is identical to the procedures in place for our previous study of *Selective Computed Head Tomography in Blunt Trauma* (PHS Grant No. R01 HS09699).

**5.2 - Adequacy of protection against risk**

The study poses no medical risk to patients, and the following actions will be taken to prevent any risk related to the confidentiality of medical records. Study index numbers will be used to link radiographic results and the need for neurosurgical intervention to survey data at all sites. At the time radiographic imaging is requested the patients medical record number will be recorded along with the study index number in an encoded file that will be available only to study research personnel. The information in this linkage file will be used to connect survey data with outcome data. The linkage files will be destroyed, after quality assurance reviews are complete and the study data is finalized.

**5.3 - Potential benefits of the proposed research to the subjects and others**

The proposed research will not benefit the research subjects but will provide benefit to others. In particular, this proposal seeks to reduce computed head tomography by validating the ability of a previously developed decision instrument to reliably identify blunt head injury victims who have no risk of significant intracranial injury, and who can subsequently be safely spared tomographic imaging. Validation of the decision instrument would help reduce the human and economic losses associated with over utilization of CT scanning.

**5.4 - Importance of knowledge to be gained**

The knowledge gain from this research will enable clinicians to use tomographic imaging more effectively. It will also provide substantial information on the demographics of head injury including overall prevalence, geographic and seasonal variations, and distributions by age, race and sex. Tomographic reports will yield important information on the different types of intracranial injuries, including prevalence and distributions among the various demographic groups and the clinical presentations of different injury patterns. This information will be of use in emergency neurological and clinical assessment, and will prove useful in formulating future recommendations. Data on the presence or absence of the criteria in individual patients will be useful in assigning clinical significance to the criteria. This data will be analyzed by regression analysis to identify relationships between intracranial injury and clinical presentation. Such studies can aid in risk assessment and stratification of risk among blunt head injury patients. This information will in turn guide future studies that seek to improve the management of blunt head trauma victims. Finally, even the failure to validate the decision instrument is an important finding on its own, should it occur. It would suggest that clinicians must be extremely careful in evaluating head injury patients and that tomographic scanning may be indicated in many if not most of these individuals. These benefits will accrue without significant risk to the study population.

**5.6 - Inclusion of women**

The sites participating in the study are expected to enroll a diverse population containing all races and both sexes. Women will be enrolled in the study in the same proportions as they present with blunt head injuries. The study poses no special risk to women, and there are no special circumstances that apply to their participation. The information collect by the study will be analyzed to determine whether sex plays an important role in the identification of blunt head injuries.

**5.7 - Inclusion of minorities**

The sites participating in the study are expected to enroll a diverse population containing all races and both sexes. Minorities will be enrolled in the study in the same proportions as they present with blunt head injuries. The study poses no special risk to minorities, and there are no special circumstances that apply to their participation. Study sites have been carefully chosen to insure a broad enrollments and a diverse population. The information collect by the study will be analyzed to determine whether race plays an important role in the identification of blunt head injuries.

**5.8 - Inclusion of children**

The sites participating in the study are expected to enroll a diverse population containing all ages. Children will be enrolled in the study in the same proportions as they present with blunt head injuries. The study poses no special risk to children, and there are no special circumstances that apply to their participation. Study sites have been carefully chosen to insure a broad enrollments and a diverse population. The information collect by the study will be analyzed to determine whether age plays an important role in the identification of blunt head injuries.

**5.9 - Data and safety monitoring plan**

Not applicable, this is not a Phase I, II, or III clinical trial.

**6. LITERATURE CITED**

1 Levitt MA. Minor head injury. Ann Emerge Med 1994;23:1143-4.

2 Stiell IG, Wells GA, Vandemheen K, et al. Variation in ED use of computed tomography for patients with minor head injury. Ann Emerg Med 1997;30:14-22.

3 Graham ID, Stiell IG, Laupacis A, O’Connor AM, Wells GA. Emergency physicians’ attitudes toward and use of clinical decision rules for radiography. *Acad Emerg Med* 1998;5:134-40.

4 Fischer RP, Carlson J Perry JF. Postconcussive hospital observation of alert patients in a primary trauma center. *J Trauma* 1981;21:920-4.

5 Jones JJ, Jeffreys. Relative risk of alternative admission policies for patients with head injuries. *Lancet* 1981;2:850-2.

6 Rimel RW, Giordani B, Barth JT, Boll TJ, Jane JA. Disability caused by minor head injury. *Neurosurgery* 1981;9:221-8.

7 Rimel RW, Giordani B, Barth JT, Jane JA. Moderate head injury: completing the clinical spectrum of brain trauma. *Neurosurgery* 1982;11:344-51.

8 Marshall LF, Toole BM, Bowers SA. The National Traumatic Coma Data Bank: Part 2: Patients who talk and deteriorate: Implications for treatment. *J Neurosurg* 1983;59:285-8.

9 Rockswold GL, Leonard PR, Nagib MG. Analysis of management in thirty-three closed head injury patients who “talked and deteriorated.” *Neurosurgery* 1987;21:51-5.

10 Leclercq TA, Bolton R. Indications for CT scanning in benign head trauma. *Rhode Island Med J* 1985;68:503-5.

11 Dacey RG, Alves WM, Rimel R, et.al. Neurosurgical complications after apparently minor head injury: assessment of risk in a series of 610 patients. *J Neurosurg* 1986;65:203-10.

12 Feuerman T, Wackym PA, Gade GF, et. al. Value of skull radiography, head computed tomographic scanning and admission for observation in cases of minor head injury. *Neurosurgery* 1988;22:449-53.

13 Rivara F, Tanaguchi D, Parish RA, Stimac GK, Mueller B. Poor prediction of positive computed tomographic scans by clinical criteria in symptomatic pediatric head trauma. *Pediatrics* 1987;80:579-84.

14 Klauber MR, Marshall LF, Luerssen TG, Frankowski R, Tabaddor K, Eisenberg HM. Determinants of head injury mortality: importance of the low risk patient. *Neurosurgery* 1989;24:31-6.

15 Ashkenazi E, Constantini S, Pomeranz S, et. al. Delayed epidural hematoma without neurologic deficit. *J Trauma* 1990;30:613-6.

16 Stein SC, Ross SE. The value of computed tomographic studies in patients with low-risk head injuries. *Neurosurg* 1990;26:638-40.

17 Livingston DH, Loder PA, Koziol J, Hunt CD. The use of CT scanning to triage patients requiring admission following minimal head injury. *J Trauma* 1991;31:483-7.

18 Harad FT, Kerstein MD. Inadequacy of bedside clinical indicators in identifying significant intracranial injury in trauma patients. *J Trauma* 1992;32:359-63.

19 Stein SC, O’Malley KF, Ross SE. Is routine computed tomography scanning too expensive for mild head injury? *Ann Emerg Med* 1991;20:1286-9.

20 Stein CS, Ross SE. Mild head injury: a plea for routine early CT scanning. *J Trauma* 1992;33:11-3.

21 Lobato RD, Rivas JJ, Gomez, et al. Head-injured patients who talk and deteriorate into coma: analysis of 211 cases studied with computerized tomography. *J Neurosurg* 1991;75:256-62.

22 Jeret JS, Mandell M, Anziska B, et. al. Clinical predictors of abnormality disclosed by computed tomography after mild head trauma. *Neurosurgery* 1993;32:9-16.

23 Schynoll W, Overton D, Krome R, et. al. A prospective study to identify high yeild criteria associated with acute intracranial computed tomography finding in head-injured patients. *Am J Emerg Med* 1993;11:321-6.

24 Borczuk P. Predictors of intracranial injury in patients with mild head injury. *Ann Emerg Med* 1995;25:731-6.

25 Miller EC, Derlet RW, Kinser D. Minor Head Trauma: Is computed tomography always necessary? *Ann Emerg Med* 1996;27:290-4.

26 Cullotta VP, Sementilli MF, Gerold K, et. al. Clinicopathological heterogeneity in the classification of mild head injury. *Neurosurgery* 1996;38:245-50.

27 Teasdale G, Jennett B. Assessment of coma and impaired consciousness. A practical scale. *Lancet* 1974;2:81-4.

28 Moran SG, McCarthy MC, Uddin DE, Poelstra RJ. Predictors of positive CT scans in the trauma patient with minor head injury. *Am Surgeon* 1994;60:533-5.

29 Madden C, Witzke DB, Sanders AB, Valente J, Fritz M. High-yield selection criteria for cranial computed tomography after acute trauma. *Acad Emerg Med* 1995;2:248-53.

30 Fulton RL, Everman D, Mancino M, et. al. Ritural head computed tomography may unnecessarilty delay lifesaving trauma care. *Surg Gynecol Obstet* 1993;176:327-32.

31 Thornbury JR, Campbell JA, Masters SJ, et. al. Skull fracture and the low risk of intracranial sequelae in minor head trauma. AJR 1984;143:661-4.

32 Masters SJ. Evaluation of head trauma: efficacy of skull films. *AJR* 1980;135:539-47.

33 Servadei F, Ciucci G, Morichetti A, et al. Skull fracture as a predictor of increased risk in minor head injuries. *Surg Neurol* 1988;30:364-9.

34 Servadei F, Ciucci G, Pagano F, et al. Skull fracture as a risk factor of intracranial complications in minor head injuries: a prospective CT study in a series of 98 adult patients. *J Neurol Neuro Surg Psych* 1988;51:526-8.

35 Mendelow AD, Teasdale G, Jennett B, Bryden J Hessett C, Murray G. Risks of intracranial haematoma in head injured adults. *BMJ* 1983;287:1173-1176.

36 Miller JD, Murray LS, Teasdale GM. Development of a traumatic intracranial hematoma after a “minor’ head injury. *Neurosurg* 1990;27:669-73.

37 Shah AK, Guyot AM, Ham SD, Diaz FG, Peterson PL. “CT or not CT”: ER evaluation of head trauma. *Neurology* 1991;41(Suppl 1):308.

38 Taheri PA, Karamanoukian H, Gibbons K, Waldman N, Doerr RJ, Hoover EL. Can patients with minor head injuries be safely discharged home?  *Arch Surg* 1993;128:289-92.

39 Reinus WR, Wippold FJ, Erickson KK. Practical selection criteria for noncontrast cranial computed tomography in patients with head trauma. *Ann Emerg Med* 1993;22:1148-55.

40 Reinus WR, Erickson KK, Wippold FJ. Unenhanced emergency cranial CT: optimizing patient selection with univariate and multivariate analyses. *Radiology* 1993;186:763-8.

41 Mohanty SK, Thompson W, Rakower S. Are CT scans for head injury patients always necessary*? J Trauma* 1991;31:801-4.

42 Mower WR, Hoffman JR, Schriger DL. The feasibility of selective radiography in patients with trauma-induced neck pain (abstr). *Ann Emerg Med* 1990;19:1220-1.

43 Bender MB, Christoff N. Nonsurgical treatment of subdural hematomas. *Arch Neurol* 1974;31:73-9.

44 Bullock R, Smith RM, van Dellen JR. Nonoperative management of extradural hematoma. *Neurosurgery* 1985;16:602-5

45 Hamilton MG, Frizzell B, Tranmer BI. Chronic subdural hematoma: The role of craniotomy reevaluated. *Neurosurgery* 1993;33:67-72.

46 Croce MA, Dent DL, Menke PG, et al. Acute subdural hematoma: nonsurgical management of selected patients. *J Trauma* 1994;36:820-6.

47 Mikhail MG, Levitt MA, Christopher TA, Sutton MC. Intracranial injury following minor head trauma. *Am J Emerg Med* 1992;10:24-6.

48 Young KD, Lewis RJ. What is confidence? Part 2: detailed definition and determination of confidence intervals. *Ann Emerg Med* 1997;30:311-8.

49 Duus BR, Lind B, Christensen H, Nielsen OA. The role of neuroimaging in the initial management of patients with minor head injury. *Ann Emerg Med* 1994;23:1279-83.

50 Stein SC, Young GS, Talucci RC, Greenbaum BH, Ross SE. Delayed brain injury after head trauma: significance of coagulopathy. *Neurosurgery* 1992;30:160-5.

51 Report to the Chairman, Subcommittee on Health for Families and the Uninsured, Committee on Finance, U.S. Senate. **Emergency Departments Unevenly Affected by Growth and Change in Patient Use.** Washington D.C.: United States General Accounting Office, 1993 pp 4.

52 Voss M, Knottenbelt JD, Peden MM. Patients who reattend after head injury: a high risk group. *BMJ* 1995;311:1395-8.

53 Max W, MacKenzie EJ, Rice DP. Head injuries: costs and consequences. *J Head Trauma Rehabil* 1991;6:76-91.

54 Nishizawa K, Maruyama T, Takayama M, et al. Determinants of organ doses and effective dose equivalents from computed tomographic examination. *Br J Rad* 1991;64:20-8.

55 National Research Council, Committee on the biological effects of ionizing radiations (BIER V). **Health Effects of Exposure to Low Levels of Ionizing Radiation.** Washington D.C.: National Academy Press, 1990, pp 242-351.

56 Centers for Disease Control and Prevention. Abridged life table for the total population, 1992. *Monthly Vital Statsitics Report* 1995;43(6S):18.

57 Huda W, Sandison GA. CT dosimetry and risk estimates. *Radiation Protection Dosimetry* 1985;12:241-9.

58 Fleiss JL. **Statistical Methods for Rates and Proportions, ed 2.** New York, J Wiley & Sons, 1981, pp 212.

59 Suarez-Almazor ME, Belseck E, Russell AS, Mackel JV. Use of lumbar radiographs for the early diagnosis of low back pain. *JAMA* 1997;277:1782-6.

60 Mahadevan S, Mower WR, Hoffman JR, Peeples N, Goldberg W, Sonner R. Interrater reliability of cervical spine injury criteria in patients with blunt trauma. *Ann Emerg Med* 1998;31:197-201.

61 Hoffman JR, Mower WR, Wolfson A, Knox T. Selective cervical spine radiography in blunt trauma. *Ann Emerg Med* In Press.

1. Haydel MJ, Preston CA, Mills TJ, Luber S, Bladeau E, DeBlieux PMC. Indications for computed tomography in patients with minor head injury. *N Engl J Med* 2000;343:100-05.
2. Stiell IG, Wells GA, Vandemheen K, Clement C, Lesuik H, Laupacis A, *et al.* The Canadian CT head rule for patients with minor head injury. *Lancet* 2001;357:1391-6.
3. Marx JA, Biros MH. Who is at risk after head or neck trauma? *N Engl J Med* 2000;343:138-40.
4. Mower WR, Hoffman JR, Herbert M, Wolfson AB, Pollack CV, Zucker MI; NEXUS II Investigators. Developing a clinical decision instrument to rule out intracranial injuries in patients with minor head trauma: methodology of the NEXUS II investigation. *Ann Emerg Med* 2002;40:505-14.
5. Stiell IG, McKnight RD, Greenberg, *et al*. Implementation of the Ottawa Ankle Rules. *JAMA* 1994;271:827-32.
6. Stiell IG, Wells GA, Hoag RA, Sivilotti MLA, Cacciotti TF, Verbeek RP,  *et al*. A multicenter trial to introduce clinical decision rules for the use of radiography in acute ankle injuries. *BMJ* 1995;311:594-7.
7. Shackford SR, Wald SL, Ross SE, *et al*. The clinical utility of computed tomographic scanning and neurologic examination in the management of patients with minor head injuries. *J Trauma* 1992;33:385-94.
8. Stein SC, Ross SE. Minor head injury: a proposed strategy for emergency management. *Ann Emerg Med* 1993;22:1193-96.
9. **Advanced trauma life support instructor manual, ed 5**. Chicago, American College of Surgeons. 1993.
10. Hollander JE, Go S, Lowery DW, Wolfson AB, Pollack CV, Herbert M, Mower WR, Hoffman JR. Inter-rater reliability of criteria used in assessing blunt head injury patients for intracranial injuries. *Acad Emerg Med* 2003;10:830-5*.*
11. Atzema C, Mower WR, Hoffman JR, Holmes JF, Killian AJ, Wolfson AB, Krawczyk J. Defining “clinically unimportant” CT findings in patients with blunt head trauma. *Acad Emerg Med* 2002;9:451.
12. Efron B, Tibshirani RJ. **An Introduction to the Bootstrap**. Boca Raton, Florida, Chapman and Hall/CRC, 1998.
13. Mower WR, Hoffman JR, Herbert M, Wolfson AB, Pollack CV, Zucker MI; NEXUS II Investigators. Developing a decision instrument to guide computed tomographic imaging of blunt head injury patients. *J Trauma* 2005;59:954-9.

**Appendix A – Participating Centers**

| Site Investigators/  Institutions: | 1 - Ronald Reagan UCLA Medical Center  757 Westwood Plaza  Los Angeles, CA 90095  University based private hospital  Site Liaison - William R. Mower, MD, PhD  UCLA – Emergency Medicine Center  924 Westwood Blvd., Suite 300  Los Angeles, CA 90024  Phone number: 310-794-0582  Fax number: 310-794-0599  E-mail: wmower@ucla.edu  2 - Antelope Valley Hospital Emergency Department  1600 W Avenue J  Lancaster, CA 93534  Community based private Hospital  Site Liaison - Malkeet Gupta, MD  UCLA – Emergency Medicine Center  924 Westwood Blvd., Suite 300  Los Angeles, CA 90024  and  Antelope Valley Hospital Emergency Department  1600 W Avenue J  Lancaster, CA 93534  E-mail: [malkeet@gmail.com](mailto:malkeet@gmail.com)  3 - San Francisco General Hospital  505 Parnassus Ave.  San Francisco, CA 94143  Public hospital  Site Liaison - Robert Rodriguez, MD  San Francisco General Hospital  Department of Emergency Medicine  UCSF School of Medicine  505 Parnassus Ave.  San Francisco, CA 94143  E-mail: Robert.rodriguez@ucsf.edu  4 - UCSF Fresno, Community Regional Medical Center  2823 Fresno St.  Fresno, CA 93721  Public hospital  Site Liaison - Gregory Hendey, MD  UCSF Fresno, Community Regional Medical Center  Department of Emergency Medicine  2823 Fresno St.  Fresno, CA 93721  E-mail: ghendey@fresno.ucsf.edu |
| --- | --- |

**Appendix B - Blunt Head Trauma Questionnaire**

**DEMOGRAPHICS**

Date: / / ID # _____________

Date of Birth: / / Sex: **🞎** Female **🞎** Male **🞎** Unknown

Race:

**🞎** White

**🞎** Black

**🞎** Hispanic

**🞎** Asian

**🞎** Middle Eastern

**🞎** Native American

**🞎** Other

**REASON FOR COMPUTED HEAD TOMOGRAPHY**

**🞎** Blunt Head Injury **🞎** Other

**PATIENT STATUS**

**🞎** Stable (Print request after complete data entry) **🞎** Unstable (Print request with incomplete data)

**EVALUATION**

Please indicate whether any of the following characteristics are present:

Signs of basilar, depressed or diastatic skull fracture: **🞎** Yes **🞎** No **🞎** Unknown

Scalp Hematoma: **🞎** Yes **🞎** No **🞎** Unknown

Neurologic deficit: **🞎** Yes **🞎** No **🞎** Unknown

Abnormal level of alertness: **🞎** Yes **🞎** No **🞎** Unknown

Abnormal behavior: **🞎** Yes **🞎** No **🞎** Unknown

Recurrent or forceful vomiting: **🞎** Yes **🞎** No **🞎** Unknown

Coagulopathy: **🞎** Yes **🞎** No **🞎** Unknown

If yes, check all that apply

Coumadin **🞎** Yes **🞎** No **🞎** Unknown

Aspirin/salicylate **🞎** Yes **🞎** No **🞎** Unknown

Clopidogrel **🞎** Yes **🞎** No **🞎** Unknown

Other (e.g. liver failure, hemophilia, ITP, etc.) **🞎** Yes **🞎** No **🞎** Unknown

Comorbidity (e.g. A-fib, CAD, COPD, Cancer, etc.) **🞎** Yes **🞎** No **🞎** Unknown

Eyes spontaneously open with blinking: **🞎** Yes **🞎** No **🞎** Unknown

Oriented - knows name, age, etc.: **🞎** Yes **🞎** No **🞎** Unknown

Obeys verbal commands: **🞎** Yes **🞎** No **🞎** Unknown

Amnesia > 30 minutes before injury **🞎** Yes **🞎** No **🞎** Unknown

Dangerous mechanism **🞎** Yes **🞎** No **🞎** Unknown

**Appendix C – Detailed Description of Criteria Included in the NEXUS Head CT Instrument**

Terms are defined for purposes of clarity and to ensure consistent data collection.

*Evidence of skull fracture includes* signs of *basilar skull fracture* (including, but are not limited to) periorbital or peri-auricular ecchymoses, hemotympanum, and drainage of clear fluid from the ears or nose. Signs of *depressed or diastatic skull fracture* include a palpable step-off of the skull, a stellate laceration from a point source, or any injury produced by an object striking a localized region of the skull (such as a baseball bat, club, pool cue, golf-ball, baseball, pipe, etc.).

*Scalp hematoma* refers to swelling secondary to hematoma formation over any portion of the bony calvarium. Injuries that do not involve the calvarium, including hematomas limited to the face and neck are not considered scalp hematomas.

*Neurologic deficit* refers to any abnormal neurologic finding revealed by detailed testing. This may include motor or sensory deficits (abnormal weakness or sensation in any one or more of the four extremities, as determined by systematic testing of muscle strength and sensation in all four limbs), cranial nerve abnormality (particularly cranial nerves II-XII, as determined by systematic testing of each nerve), cerebellar abnormality as manifest by ataxia, dysmetria, dysdiadokinesis, or other impairment of cerebellar function (as determined by systematic testing of cerebellar function, including tests of ataxia, and finger-nose-finger, heel-to-shin, and rapid alternating movements), gait abnormality or inability to walk normally (may be due to inadequate strength, loss of balance or ataxia; it is determined by systematic testing of gait, including tandem and heel-to-toe walking, and Romberg testing), or any other impairment of neurological function.

*Abnormal level of alertness* is evidenced by a variety of findings, including but not limited to a Glasgow coma score of 14 or less; delayed or inappropriate response to external stimuli; excessive somnolence; disorientation to person, place, time or events; inability to remember three objects at five minutes; perseverating speech; and other neurological impairments.

*Abnormal behavior* is any inappropriate action displayed by the victim. It includes such things as excessive agitation, inconsolability, refusal to cooperate, lack of affective response to questions or events, and violent activity.

*Persistent vomiting* is evidenced by recurrent (more than one episode), projectile or forceful emesis (either observed or by history) after trauma.

*Coagulopathy* is any impairment of normal blood clotting such as occurs in hemophilia, secondary to medications (Coumadin, heparin, aspirin, etc.), hepatic insufficiency and other conditions.

*Age 65 years or more* is determined by available history.

**Appendix D – Scripted Survey Form for Long-Term Follow-Up of Patients Who Do Not Undergo Tomographic Imaging During Their Initial Evaluation**

*Script for contacting study participants by telephone for long-term follow-up.*

Hello, Mr/s.__________________, my name is __________________. I work with Dr. William Mower at the UCLA Emergency Medicine Center, and we are working on a research study on head injury. Your participation is completely voluntary. If you don’t mind, I’d like to tell you a little more about the study, and what is involved in participation. Please know that it is your right to decline to participate in this study for any reason, at any time. In addition, whether your decision to participate or not will not affect any aspect of your future medical care.

The study is being done to make sure that patients who have been seen in the emergency room and who do not receive a type of x-ray called a head CT do not have any significant head injury. Your participation in this study would consist entirely of answering a few short questions, which will help us determine if you have had any problems that might have been caused by your head injury after you left the emergency room. We will not record your name or patient number, and your participation would be completely anonymous.

If you have any questions or concerns about your right as a research subject, you may contact the UCLA Office for Protection of Research Subjects. Their phone number is 310-825-7122, or you may write then at OPRS, P.O. Box 95-1694, Los Angeles, CA 90095-1694.

Would you like to take part in the study?

Do you have any questions or concerns I can address for you about the study?

(If study participant answers affirmatively to participate, then present study related questions as follows:).

Did you return to the hospital or go to another facility after your discharge from the emergency room.

**🞎** Yes **🞎** No **🞎** Unknown

If yes, did you receive any x-rays or imaging studies?

**🞎** Yes **🞎** No **🞎** Unknown

If yes, do you know if you had the following types of x-rays taken?

Head CT (CAT scan) **🞎** Yes **🞎** No **🞎** Unknown

Skull x-rays **🞎** Yes **🞎** No **🞎** Unknown

MRI or magnetic imaging **🞎** Yes **🞎** No **🞎** Unknown

Did you receive any of the following treatments:

Any treatment **🞎** Yes **🞎** No **🞎** Unknown

Admission **🞎** Yes **🞎** No **🞎** Unknown

Brain surgery **🞎** Yes **🞎** No **🞎** Unknown

Thank you so much for your participation.

(if study participant does not answer affirmatively, respond as follows:)

Thank you for your time. Good-bye.

**APPENDIX E – Specific Duties of Study Personnel**

**1 - Principal Investigator**

The principal investigator will be responsible for the overall direction of the project and its day-to-day operation. Specific duties will include:

•Working with the sites and coordinators to maximize the power and validity of the study, and develop solutions to problems arising during study execution.

•Designing and implementing the computer support systems and databases.

•Supervising the configuration of the data collection system.

•Training and orienting the site liaisons and coordinators in study protocols and the data collection system.

•Working with the site liaisons and coordinators to ensure effective implementation of study protocols throughout all involved institutions.

•Supervising the project steering committee.

•Supervising the research assistant and overseeing the collection, transmission, concatenation and analysis of data.

•Working with the co-investigators to complete the statistical and validity analyses. Working with the co-investigators to complete all study reports.

•Working with UCLA Emergency Medicine administrators and accountants to ensure adherence to the budget.

**2 - Site Liaisons**

The site liaisons will be charged with:

•Hiring and supervising the study coordinator in their site.

•Working with the Principal Investigator in trouble-shooting and correcting any protocol problems, and maximizing the validity of the study.

•Working to ensure effective implementation of study protocols at their institution.

•Obtaining any institutional authorizations needed for study protocol enactment at each site

•Enlisting the assistance of all emergency physicians and radiologists at the site.

•Performing random quality assurance checks of physician examinations and data collection to ensure adherence with study protocols.

•Serving on the project steering committee.

•Assisting site coordinators in performing quality assurance checks by obtaining access to the radiographic reports, radiographic logs, quality assurance rosters, patient records and discharge logs.

**3 - Site Coordinators**

Specific duties of the site coordinators will include:

•Collecting copies of all computed head tomograph reports, and forwarding them to UCLA.

•Assisting in trouble-shooting and correcting software and database problems at their sites.

•Performing quality assurance checks on the data entered into the database by verifying that physicians complete a computer record for every blunt head injury patient undergoing computed head tomography and that all radiographic reports are recovered and entered into the study database.

•Reviewing discharge logs and radiology logs to detect any intracranial injuries that may have been missed on initial screening.

**4 - Site Physicians**

The site Emergency Physicians will be responsible for:

•Evaluating patients and ordering radiographic studies as defined by the protocol.

•Assessing and recording survey data on all blunt trauma patients for whom they order CT head imaging.

Site Radiologists will be responsible for:

•Interpreting radiographs and reporting their findings.

•Ensuring that x-ray technicians perform computed tomographic head imaging only after survey data is collected and recorded.

**5 - Research Assistant**

The research assistant will maintain the central UCLA database. Duties will include:

•Reviewing and concatenating survey data received form each of the study sites.

•Abstracting and concatenating radiographic reports and survey data.

•Assisting in software updates and data analysis.

**6 - Clinical Research Assistants (Ronald Reagan UCLA Medical Center)**

The clinical research assistants at the Ronald Reagan UCLA Medical Center will identify and enroll blunt head injury patients for the verification bias section of the study. Their duties will include:

•Working with emergency physicians to identify, enroll and obtain informed consent for the follow-up survey from all emergency department patients who have sustained blunt head injury.

•Identifying the preferred time and number for the three-month telephone follow-up survey of each enrolled patient.

•Scheduling interview times to perform three-month follow-up surveys on patients who lack telephone access.

•Contacting patients or their caregivers at the previously agreed time and location to perform three-month follow-up interviews.

•Entering survey information into the computerized database.

•Performing quality assurance checks to verify that all blunt head injury patients are enrolled and surveyed.

•Immediately notifying the Regional Administrator of any patients who required neurosurgical interventions, but were not imaged during their initial presentation.
